# Whole genome DNA sequencing provides an atlas of somatic mutagenesis in healthy human cells and identifies a tumor-prone cell type

**DOI:** 10.1186/s13059-019-1892-z

**Published:** 2019-12-18

**Authors:** Irene Franco, Hafdis T. Helgadottir, Aldo Moggio, Malin Larsson, Peter Vrtačnik, Anna Johansson, Nina Norgren, Pär Lundin, David Mas-Ponte, Johan Nordström, Torbjörn Lundgren, Peter Stenvinkel, Lars Wennberg, Fran Supek, Maria Eriksson

**Affiliations:** 1 Department of Biosciences and Nutrition, Center for Innovative Medicine, Karolinska Institutet, Huddinge, Sweden; 2 Department of Medicine Huddinge, Integrated Cardio Metabolic Center, Karolinska Institutet, Huddinge, Sweden; 3 Science for Life Laboratory, Department of Physics, Chemistry and Biology, Linköping University, Linköping, Sweden; 4 Science for Life Laboratory, Department of Cell and Molecular Biology, Uppsala University, Uppsala, Sweden; 5 Science for Life Laboratory, Department of Molecular Biology, Umeå University, Umeå, Sweden; 6 Science for Life Laboratory, Department of Biochemistry and Biophysics (DBB), Stockholm University, Stockholm, Sweden; 7 Genome Data Science, Institute for Research in Biomedicine (IRB Barcelona), The Barcelona Institute of Science and Technology, 08028 Barcelona, Spain; 8 Department of Clinical Sciences, Intervention and Technology, Karolinska Institutet, Division of Transplantation Surgery, Karolinska University Hospital, Huddinge, Sweden; 9 Department of Clinical Sciences, Intervention and Technology, Karolinska Institutet, Division of Renal Medicine, Karolinska University Hospital, Huddinge, Sweden; 10 Institució Catalana de Recerca i Estudis Avançats (ICREA), Barcelona, Spain

**Keywords:** Somatic mutations, Aging, Kidney cancer, Proximal tubule, kidney progenitors

## Abstract

**Background:**

The lifelong accumulation of somatic mutations underlies age-related phenotypes and cancer. Mutagenic forces are thought to shape the genome of aging cells in a tissue-specific way. Whole genome analyses of somatic mutation patterns, based on both types and genomic distribution of variants, can shed light on specific processes active in different human tissues and their effect on the transition to cancer.

**Results:**

To analyze somatic mutation patterns, we compile a comprehensive genetic atlas of somatic mutations in healthy human cells. High-confidence variants are obtained from newly generated and publicly available whole genome DNA sequencing data from single non-cancer cells, clonally expanded in vitro. To enable a well-controlled comparison of different cell types, we obtain single genome data (92% mean coverage) from multi-organ biopsies from the same donors. These data show multiple cell types that are protected from mutagens and display a stereotyped mutation profile, despite their origin from different tissues. Conversely, the same tissue harbors cells with distinct mutation profiles associated to different differentiation states. Analyses of mutation rate in the coding and non-coding portions of the genome identify a cell type bearing a unique mutation pattern characterized by mutation enrichment in active chromatin, regulatory, and transcribed regions.

**Conclusions:**

Our analysis of normal cells from healthy donors identifies a somatic mutation landscape that enhances the risk of tumor transformation in a specific cell population from the kidney proximal tubule. This unique pattern is characterized by high rate of mutation accumulation during adult life and specific targeting of expressed genes and regulatory regions.

## Background

Over a lifetime, the human body is vulnerable to a vast number of mutagenic forces that collectively lead to loss of genome integrity and subsequently cellular aging and cancer initiation [1]. Sequencing studies have revealed genetic variations among cells within an individual, referred to as “somatic variance.” This information can be used to study the genome evolution during the lifespan of an individual [2] and outline specific mutagenic processes that promote the transition from a normal to a cancer cell [3]. Variants that are exclusively detected in the clonal-cell population of a tumor are believed to represent the mutations that occurred in the cell prior to the initiation of cancer [4] and are widely used to study mutational processes in normal tissues. However, inherent within cancer clones are characteristics (increased genomic instability and selective advantage), which can present a conundrum in understanding the etiology of somatic mutations in normal tissues. The elimination of confounding factors can be achieved by studying mutations in *non-cancerous cells*, thus allowing a direct assessment of genomic changes occurring with typical aging of organ systems. Whole genome sequencing (WGS) of a high number of single cells would be the most informative method. However, there are technical challenges associated with single-cell WGS and these have impeded massive analysis of somatic variance in normal cells [5, 6]. An alternative strategy is the bulk sequencing of non-cancer human tissues [7–10]. This approach provides only selected variants, i.e., variants contained in the genome of cells that clonally expanded in the normal tissues and contributed a detectable number of copies. But, similar to what observed for cancer, detectable variants may not be fully representative of the common mutational processes. In addition, bulk data are not ideal for analyses that compare the frequency of mutations in specific genomic regions or for exploring the non-coding portion of the genome [7–10]. It is possible to obtain WGS data relative to a single genome while avoiding single cell sequencing. This method requires in vitro clonal expansion of a single cell prior to sequencing, and a specific processing of data, in order to select the somatic variants that were present in vivo and eliminate those that occurred during culture [2, 6]. This strategy has some limitations. For example, it is necessarily restricted to cells that are able to proliferate in vitro (e.g., stem/progenitor cells or reprogrammed cells), and the culturing procedure is demanding and not suitable for the analysis of a large number of cells. Despite these limitations, the strategy has been successfully applied to the analysis of skeletal muscle progenitors [11]; intestine, colon, and liver stem cells [12]; blood stem and progenitor cells [13, 14]; and reprogrammed skin fibroblasts [15].

Results generated from clonally expanded, normal cells demonstrate that aging is correlated with a linear increase of somatic mutations and specific mutation patterns and distributions. These features appear very consistent among different cells of the same tissue, even when obtained from different individuals. Therefore, despite the low number of genomes analyzed per tissue, important general conclusions regarding the rate of occurrence and the main features of somatic mutations have been drawn for skeletal muscle, liver and intestinal stem cells, and blood cells during aging [11, 12, 14]. Importantly, information can be gleaned from these data and used to build an understanding of cellular and genomic activities prior to the appearance of mutations. A catalogue of somatic mutations can be deconstructed into distinct components or mutational signatures, through non-negative matrix factorization (NMF) [16]. In multiple cases, mutational signatures obtained through the analysis of thousands of cancer genomes have efficiently been attributed to a specific etiology [17] (http://cancer.sanger.ac.uk/cosmic/signatures). This is the case of signature 7, which is found predominantly in cancers derived from the skin and is consistent with the chemical modifications of DNA expected after sunlight UV exposure [17]. Unfortunately, the mechanisms underlying other signatures remain unknown. For example, the single base substitution signature (SBS)40 was recently separated from signature 5 and shown to induce a large number of mutations in cancer samples, especially those derived from the kidney [18]. While the etiology of signature 5 seems to be related to uncorrected errors [19, 20], the etiology of SBS40 is unexplored. Another strategy to identify the mutagens that shape a given genome is to study regional differences in the distribution of somatic mutations [21]. Genomic features that determine the non-random localization of mutations are (1) DNA replication timing [22], (2) chromatin organization [11, 23, 24], and (3) the levels of active transcription [25]. Consequently, these features influence DNA exposure to both extrinsic (genotoxic compounds and radiations) and intrinsic (DNA synthesis and repair mechanisms) mutagens [21–23, 25] and are thought to be dependent on the organ or tissue. Taken together, it is the current belief that the development of somatic mutations in healthy tissues occurs as tissue-specific somatic mutagenesis [12, 14, 17, 26].

The findings derived from our atlas of somatic mutations in healthy tissues do not support a simple association of each tissue to a specific somatic mutation pattern. In contrast, we identify a stereotypical, mutational pattern across progenitor cells from a variety of tissues and two distinct mutation profiles in the same tissue portion, indicating that mutagen exposure is modulated by multiple factors in addition to tissue type. In particular, we identify cell differentiation state and cell-type-specific activities as critical determinants of mutagenesis. Importantly, our high coverage WGS data allowed us to define that the landscape of somatic mutations in different cell types is different in terms of mutational signatures, but also genomic distribution of mutations. Our analyses, based on single genome data from the kidney, skin, subcutaneous, and visceral fat cells from healthy donors, and complemented with a meta-analysis of somatic mutations from healthy (*N* = 161) and tissue-matched cancer genomes (*N* = 192), identify a unique mutation pattern in a population of proximal tubule (PT) cells. This population expresses the distinguishing markers of a PT cell type previously identified as the cell of origin of the most common kidney cancer subtypes [27]. Its unique mutation pattern is characterized by high rate of mutation acquisition during adult life and mutation enrichment in regulatory regions and expressed genes, ultimately resulting in a higher risk of a transition to cancer. Overall, our work constitutes the proof of principle for exploiting somatic mutation data from healthy cells to tailor cell-type-specific approaches of cancer prevention.

## Results

### Detection of mutations in different tissues from the same individual

To explore differences in mutagenic processes occurring in adult human tissues, we analyzed the somatic variation in human kidney tubules (KT), epidermis (EP), and subcutaneous and visceral adipose tissue (SAT and VAT, respectively) from healthy individuals of different ages. These tissues are subjected to extensive morphological changes during aging, including loss of regenerative potential and atrophy in the case of kidney tubules, epidermis, and subcutaneous fat and progressive hypertrophy in the case of visceral fat [28, 29]. Genomic alterations, for example those connected with premature-aging syndromes, have been associated to kidney, skin, and fat changes [30–32], and our analysis aims to better establish a link between loss of genome integrity and specific morphological modifications in these tissues.

**Fig. 1:**
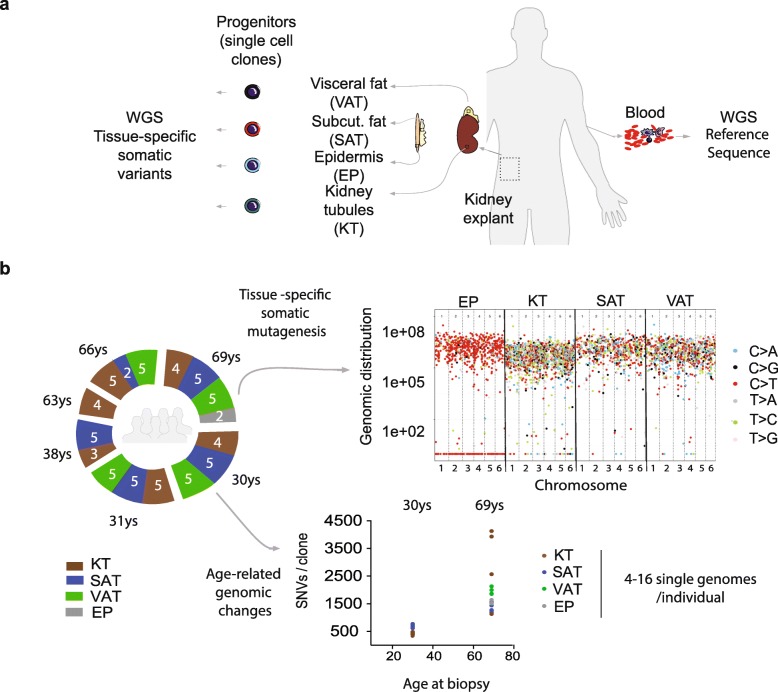
Somatic mutation detection in single genomes from different tissues of the same individual. **a** Experimental strategy for single genome analysis of progenitor cells from multiple tissues from the same healthy individual. Blood, kidney, subcutaneous fat (SAT), visceral fat (VAT), and skin biopsies were obtained from living kidney donors undergoing surgery. The blood tissue was whole genome sequenced (WGS) as a bulk to obtain the individual’s reference sequence. The kidney tubule (KT) and epidermis (EP) portions were separated from the kidney and skin biopsies, respectively. Single progenitor cells were isolated from KT, SAT, VAT, and EP and clonally expanded in culture to obtain WGS data. These data were filtered using the individual’s reference sequence to obtain the catalogue of somatic variants for every clone. **b** Schematic summary of sequenced samples and analysis strategy. Two to five single genomes per biopsy were sequenced (white numbers in the round plot) from six individuals of either younger (30–38) or older (63–69) age. KT progenitors were sequenced for all six individuals, while SAT, VAT, and EP progenitors were sequenced in a subset of the donors. Somatic mutation data were used to study either the tissue or the age effect on mutation accumulation. An example of tissue-related differences found in the study is provided (top right): somatic SNVs found in 4 clones from different tissues of the same individual were plotted according to their genomic position and in different colors according to the type of base substitution. An example of age-related changes is provided (bottom right): total amount of SNVs in the genome of each sequenced clone from two selected individuals of either younger (30 years) or older (69 years) age

Genomic data were obtained by WGS of single cells freshly isolated from tissue biopsies and clonally expanded in vitro (Fig. 1a). This strategy allowed the survey of ~ 92% of the genome at a minimum coverage of 15x and the discovery of somatic mutations present in the single cell at the moment of isolation from the tissue. A stringent filtering on the allele frequency (AF), allowing only variants with AF comprised between 0.4 and 0.6, efficiently discarded somatic variants acquired during in vitro culture (see the “Methods” section). A well-controlled comparison of tissue-specific differences was achieved through the analysis of cells derived from multiple tissues from the *same* individual (Fig. 1a, b). Multi-tissue biopsies were obtained from three living, kidney donors of younger age (30, 31, 38 years) and three donors of older age (63, 66, 69 years). Characteristics of the donor pool were as follows: (1) provided an extensive, clinical evaluation before surgery; (2) no history of cancer, only two donors reported forms of benign hyperplasia that are very common in the population; (3) a body mass index ranging from 20 to 30 kg/m^2^; and (4) normal kidney function (Additional file 1: Table S1A). None of the donors carried a genetic predisposition to cancer, according to our analysis of germline mutations in 47 known cancer genes (Additional file 1: Table S1B).

Specific cell types were cultured from all tissues tested: kidney tubule cells from the kidney, pre-adipocytes from fat, and keratinocytes from the skin (Additional file 1: Figure S1). Cells were sequenced only if they were able to attach and proliferate as a colony for 17–20 divisions (Additional file 1: Table S1C). Based on these unique properties of colony formation and long-term proliferation, we named our samples as *progenitors* from KT, EP, SAT, and VAT.

Our newly generated data comprises a total of 69 single genomes (Fig. 1b, Additional file 1: Table S1D). From one donor (a 69-year-old woman), we obtained multiple, progenitor clones from four tissues. From the other individuals, we sequenced multiple KT clones and, in most cases, also multiple SAT and VAT clones (Fig. 1b). The sequencing data yielded information on single nucleotide variants (SNVs) and small insertion/deletions (InDels) (Additional file 1: Table S1D and Additional file 2) that were validated using a technical replicate. The validation rate was 99 and 97% for SNVs and InDels, respectively (Additional file 1: Table S1E). This validation confirmed that our pipeline could recover a set of high-confidence somatic variants and exclude variants that occurred during cell culture, as demonstrated in our previous publication [11]. The false-negative rate is also expected to be the same (0.41) [11].

The data have been used in either tissue- or age-focused analyses in order to explore both the tissue-specific differences of somatic mutation accumulation and the age-related genome modifications common among tissues (Fig. 1b).

### The tissue of origin of a cell is not the only determinant of the somatic mutation profile

**Fig. 2:**
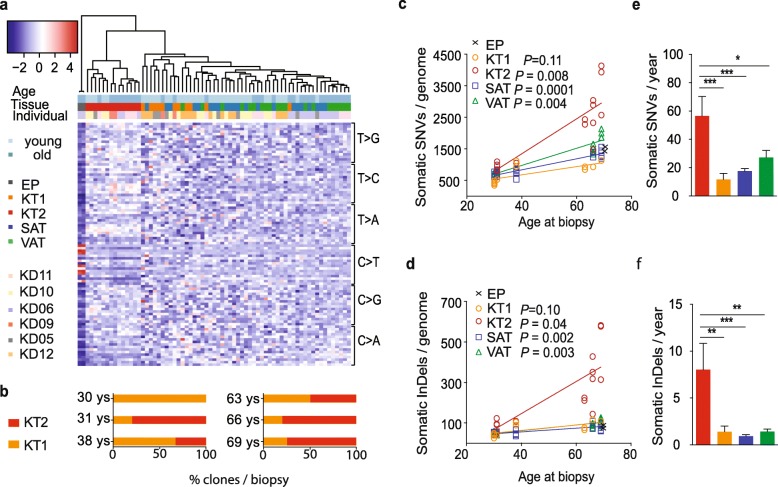
Clustering of samples on the base of mutation types defines similarities between different tissues and two subsets of KT cells. **a** Mutation pattern of 69 single genomes obtained from different human tissues of six healthy individuals of either younger (30–38) or older (63–69) age (horizontal). SNVs were subdivided in 96 classes based on the single base substitution types and their trinucleotide context (vertical) and the relative amount of mutations for each class were plotted as a heatmap. Hierarchical clustering of the samples based on the mutation pattern is shown on top of the heatmap. **b** Percentage of kidney-tubule-derived cells clustering in the KT1 or KT2 subset per biopsy. Each biopsy is defined by the age of the donor (30 years *N* = 4; 31 years *N* = 5; 38 years *N* = 3; 63 years *N* = 4; 66 years *N* = 5; 69 years *N* = 4 clones). **c**, **d** Number of somatic single nucleotide variants (SNVs, **c**) and small insertions/deletions (InDels, **d**) found in single genomes of multiple progenitors from 6 individuals of different ages. (*x* axis) The numbers of somatic variants per clone were normalized to the percentage of autosomes covered by the sequencing. Linear regression curves and *P* values calculated with the linear mixed models are shown for each tissue. **e**, **f** Average yearly increase of somatic SNVs (**e**) and InDels (**f**) per tissue. * *P* < 0.05, **P < 0.01, ***P < 0.001, one-way ANOVA and multiple comparisons tests. EP epidermis, KT1 kidney tubule 1, KT2 kidney tubule 2, SAT subcutaneous fat, VAT visceral fat

To understand somatic mutagenesis in different tissues, we compared the spectrum of somatic mutations recovered in each sample. Somatic SNVs were organized in 96 classes based on the type of base substitution and its trinucleotide context. This classification yielded a somatic mutation profile that was used to cluster samples (Fig. 2a). As expected, EP samples, rich with UV-induced C > T transitions, separated from all the others (first cluster to the left). Unexpectedly, the other samples did not cluster according to the tissue of origin, but created two main subgroups. The largest group (right) included all SAT and VAT clones and some of the KT samples (KT1). The other cluster (center) consisted of the remaining KT samples (KT2; 54% of KT clones). All but one biopsy showed the concomitant presence of KT1 and KT2 cells (Fig. 2b). The KT2-mutation profile characterized all the clones with the highest numbers of variants, both SNVs and InDels (Fig. 2c, d, respectively). In agreement, KT2 clones showed higher, yearly increase of mutations (56.6 SNVs and 8.0 InDels per genome per year), compared to the other cell types (KT1 clones 11.7 SNVs and 1.4 InDels; SAT 17.5 SNVs and 0.9 InDels; VAT 27.2 SNVs and 1.4 InDels) (Fig. 2e, f).

In summary, we identify a stereotyped mutation spectrum in multiple, different tissues (KT, SAT, VAT) and two distinct spectra in the same tissue (KT1 and KT2), suggesting that the tissue of origin is not the main determinant of somatic mutation accumulation in this sample set.

### An atlas of somatic mutagenesis in healthy tissues distinguishes basal and mutagen-driven processes

**Fig. 3:**
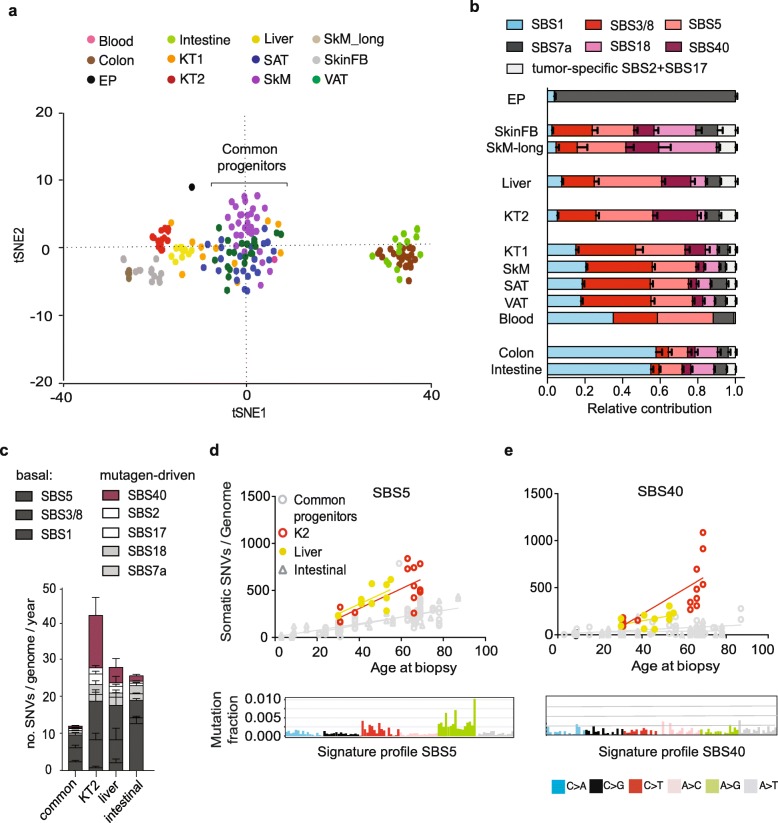
Meta-analysis of somatic mutation data from healthy donors defines basal and mutagen-driven mutagenesis in adult tissues. Sixty-nine single genomes from epidermis (EP), kidney tubule 1 (KT1), kidney tubule 2 (KT2), subcutaneous fat (SAT), and visceral fat (VAT) were analyzed together with public datasets of somatic mutations from WGS of clonally expanded non-cancer cells, including skin fibroblasts (SkinFB) [15]; liver, intestine, and colon stem cells [12]; skeletal muscle progenitors (SkM) [11]; and blood progenitors [13]. **a** tSNE plot of the trinucleotide profile of somatic SNVs. Multiple tissues displaying a common mutation profile (SkM, SAT, VAT, KT1, and blood) were named “common progenitors.” **b** Relative contribution of the eight mutational signatures identified in healthy cells via non-negative matrix factorization. Each signature was named after the most similar single base substitution (SBS) signature from [18]. **c** Average yearly increase of somatic SNVs obtained by linear fit of mutations with age in the common progenitors, KT2, liver stem cells, and intestinal stem cell (intestine and colon) groups. *P* values from linear mixed models are shown in Additional file 1: Table S3a. **d**. **e** Linear increase of mutations with age and signature profile of SBS5 (**d**) and SBS40 (**e**) in KT2 (red), liver (yellow), and common progenitors and intestine-derived (colon and intestine stem cells) samples (gray). SBS5 and SBS40 showed similar profiles (bottom), but different tissue distribution

In order to build a more comprehensive atlas of somatic mutation landscapes in human tissues, we extended our analysis to public datasets of somatic mutations from WGS of clonally expanded non-cancer cells. The cell types in this meta-analysis include skin fibroblasts (SkinFB) [15]; stem cells from the liver, intestine, and colon [12]; and progenitor cells from skeletal muscle (SkM) [11] and blood [13] (Additional file 1: Table S2). A total of 92 genomes were analyzed, in addition to our 69 genomes, and the samples subjected to unsupervised clustering on the base of their trinucleotide spectra (Fig. 3a). The groups defined in our initial clustering (Fig. 2a) were mostly maintained. Interestingly, the cluster including cells from multiple tissues (KT1, SAT, VAT) was confirmed and two more cell types, the SkM and blood progenitors, overlapped with it in the center of the plot. This cluster was called the “common progenitors” (Fig. 3a).

To understand the main factors driving the sample clustering (Fig. 3a), mutational signatures were analyzed (Fig. 3b–d and Additional file 1: Figure S2–S5). To increase the power, the WGS of 192 tissue-matched tumor samples were analyzed along with the 161 healthy samples (Additional file 1: Table S2). Eight signatures were obtained by NMF and named after the most similar, single base substitution (SBS) signature from the catalogue of signatures observed in cancer [18] (Additional file 1: Figure S2). The relative exposure of each signature in different normal and cancer types was analyzed in order to identify cell types with significantly higher exposure to specific signatures (Additional file 1: Figure S3 and Table S3). Two signatures, SBS2 (APOBEC) and SBS17b, appeared largely tumor-specific in the sample set examined here and were found at high levels in sparse cancer genomes and at negligible levels in healthy samples (Additional file 1: Figure S3). Apart from these signatures, the somatic mutation profiles found in cancer samples broadly supported the results found in the corresponding healthy samples (Additional file 1: Figure S3 and S4a).

Overall, our analysis shows that signatures SBS1, 3/8, and 5 were found ubiquitously (Additional file 1: Figure S3) and linearly increased with age (Additional file 1: Table S4). The common progenitors (SAT, VAT, KT1, SkM, and blood) presented the lowest yearly increase of mutations among the cell types analyzed, and the majority of these mutations could be attributed to SBS1, SBS3/8, and SBS5 (Fig. 3c). These evidences suggest that the signature combination comprised of SBS1, SBS3/8, and SBS5 is the unavoidable product of core cellular processes. Therefore, we define it as “basal mutagenesis.” Consistent with this concept, cell types that were not common progenitors had higher exposure to additional signatures that are associated with specific, mutagen exposure. Examples are (1) EP samples showing high levels of SBS7a, a signature induced by UV light exposure, and (2) the SkM cells used as a control for culture-induced mutagenesis in our previous study [11] (SkM-long), which showed SBS18, a signature linked to in vitro culture stress [20, 33] and consequent production of intracellular reactive oxygen species [34] (Fig. 3b). These samples were used as positive controls for prolonged exposure to a mutagen.

KT2 and liver stem cells generated two specific clusters, adjacent to each other (Fig. 3a). This similarity matched the higher rate of age-related accumulation of SBS5 seen in KT2 and liver samples (Fig. 3d). However, this increase did not seem to be the consequence of a major defect of nucleotide excision repair (NER) [19] because SBS5 was 15-fold lower in liver and KT2 cells compared to our positive controls for NER deficiency, the *ERCC2*-null tumors (Additional file 1: Figure S4b-c). In contrast to SBS5, SBS40 increased with aging mainly in KT2 cells (Fig. 3c, e). Among analyzed samples, SBS40 was stronger in KT2 and two types of kidney cancer, clear cell and papillary renal cell carcinomas (KIRC and KIRP, respectively) (Additional file 1: Figure S3). Like KT2, these tumor types demonstrated a rise in SBS40 with aging (Additional file 1: Figure S4d-e), suggesting that signature SBS40 is the result of a mutagen active in the kidney. Interestingly, the chromophobe subset of kidney carcinoma (KICH) and KT1 showed low SBS40 contribution (Additional file 1: Figure S3 and S4d-e), indicating that only specific subsets of kidney cells are exposed to the mutagenic process eliciting this signature. To obtain insight into possible mutagens active in these cells, the mutation profiles of 161 normal and 192 tissue-matched tumor samples were compared to the spectrum induced by 53 genotoxic compounds in a clonal population of iPSCs [33]. The spectrum of mutations found in KT2 and kidney tumors KIRC and KIRP (Additional file 1: Figure S5b) was similar to that generated by exposure to formaldehyde and alkylating agents, suggesting that these specific cell types in the kidney might be exposed to these mutagens, more likely derived by endogenous chemical reactions [35].

Taken together, results indicate that a group of cells from different tissues (common progenitors) provide a model of minimal mutagenesis, which we named “basal mutagenesis.” Relative to these cells, all other cell types show signs of exposure to additional extrinsic (UV light in EP, in vitro culture stress in SkM-long), intrinsic (high SBS1, probably caused by higher proliferation rate in intestinal stem cells [12]), or endogenously produced (KT2) mutagens.

### KT2 are damaged cells from the proximal tubule

**Fig. 4:**
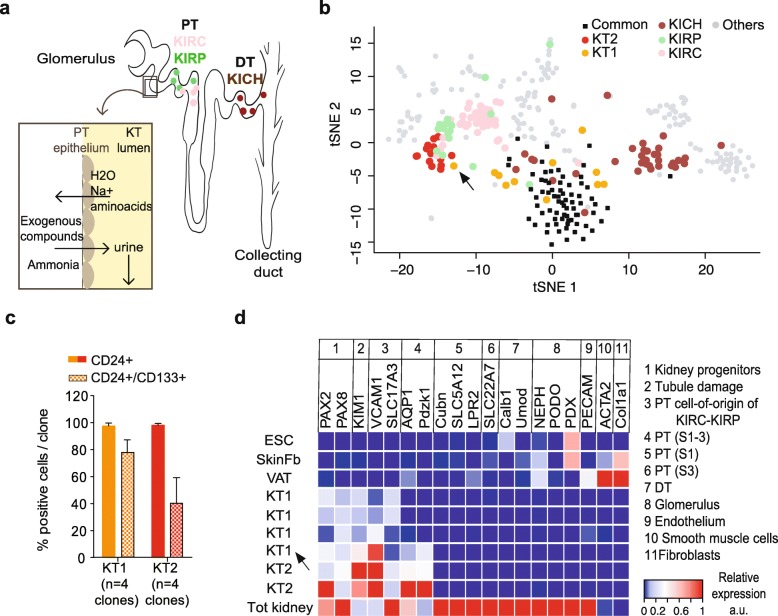
KT2 cells are proximal tubule cells exposed to mutagens. **a** Cartoon representing a kidney nephron and the location of the different tumor samples included in the analyses (according to [27, 36]). A section of proximal tubule (PT) is enlarged to show the trafficking of water, solutes, and other compounds across the PT epithelium. **b** tSNE plot of the trinucleotide profile of somatic SNVs in healthy (*n* = 161) and tumor (*n* = 192) samples. The common progenitors (SAT, VAT, SkM, and blood) and kidney-derived healthy and tumor genomes are highlighted with specific colors, while all other samples are shown in gray. **c** FACS analysis of the kidney progenitor markers CD133 and CD24 in selected KT1 and KT2 clones (*n* = 4). The average percentage of double- or single (CD24)-positive cells per clone is shown. **d** Heatmap showing the relative expression of markers of undifferentiated and differentiated kidney cells in single clones (subdivided in 11 categories described in the legend on the right) from either the KT1 (*n* = 4) or the KT2 (*n* = 2) group, tested by qPCR. Human embryonic stem cells (ESC bulk) and skin fibroblasts (SkFB bulk) were included as negative controls, together with a VAT clone. RNA extracted from a fresh kidney biopsy was included as positive control. The same KT1 clone is marked with an arrow in **b** and **d**, to highlight its intermediate KT1/KT2 phenotype at both somatic mutation (**b**) and gene expression (**d**) levels. KT1 and KT2, healthy kidney-tubule-derived cells; KIRC, clear cell renal cell carcinoma; KIRP, papillary renal cell carcinoma; KICH, chromophobe renal cell carcinoma; PT, proximal tubule; DT, distal tubule; S1, first segment of PT, convoluted; S3, last segment of PT, straight

To better understand mutagen exposure in KT cells, the similarities between normal kidney cells and different subsets of kidney cancer were further explored. A comparison of somatic mutation profiles showed that KT1 cells did not overlap with any kidney cancer type, but were intermixed with the common progenitor group (Fig. 4b). Conversely, the KT2 mutational profile was similar to KIRPs and KIRCs and very distant from the distal-tubule-derived KICH (Fig. 4b). The different subsets of kidney tumors show specific genetic, epigenetic, and transcriptional profiles [27, 36, 37], due to their origin from distinct cell types within the kidney (Fig. 4a). KIRCs and KIRPs originate from the proximal tubule (PT) [27, 36], where the epithelial layer is exposed to a continuous flow of potentially mutagenic compounds either reabsorbed from or excreted into the urine (Fig. 4a). A specific population of epithelial cells from the convoluted PT (named PT1) was recently identified as the more likely precursor of ccRCC and pRCC tumors on the base of scRNA seq data [27]. Given the similarities between KT2 and ccRCC/pRCC at the somatic mutation level, we hypothesized that KT2 clones may overlap with the PT1 population and tested the expression of a number of markers by FACS and qPCR (see the “Methods” section and Table 1). Selected KT1 and KT2 clones were tested and found positive for markers of kidney progenitors, while most markers of differentiated cells were not expressed, suggesting that both populations are in an undifferentiated state. Despite this, KT2 also expressed VCAM1/CD106 and SLC17A3, the markers that define the PT1 population found by Young et al. In addition, KT2 expressed AQP1 and PDZK1, two PT markers, and KIM1, a marker of tubule damage. The same markers were absent or expressed at lower levels in KT1 clones, except for a clone that showed a mutation spectrum very close to KT2 and alkylating agent exposure (marked with an arrow in Fig. 4a, d; Additional file 1: Figure S5b). Overall, these data suggest that KT2 cells can originate from the PT1 population, but are found in a less differentiated state. Indeed, our cell culture procedure selects for proliferating cells and KT epithelial cells are known to reacquire proliferative capacities after de-differentiation in response to tubule damage [38]. Conversely, the KT1 population expression profile is overall consistent with a previously characterized population of scattered kidney tubule progenitors [39].

**Table 1:** QPCR primers for gene expression analysis

	Forward	Reverse
ACTA2	acaggaatacgatgaagccg	gctttggctaggaatgatttgg
AQP1	ggaccggcagagctctacag	acgtcttctggacccatgct
CALB1	ttacctggaaggaaaggagctgca	tcttctgtgggtaatacgtgagcc
COL1A1	atgaccgagacgtgtggaaa	tttcttggtcggtgggtgact
CUBN	tgtttcttacggggtctgctca	gcagaccaattgcactcccttt
KIM1 (HAVCR1)	cgtgggtggttcaatgacatga	tgacggttggaacagttgtgac
LPR2	ccaaagactgttcagatgacgc	ctgagccatcatcacagtcttg
Nephrin (NPHS1)	cacacggtcagcacaacagagg	gaaacctcgggaataagacacct
PAX2	caaagttcagcagcctttcc	tcaccattggagcgaggaat
PAX8	atccggcctggagtgatagg	tggcgtttgtagtccccaatc
PDZK1	ccctgtgatgaatggaggtgt	tcatagccacaccttgaggtgt
PECAM1	ttcaagccttgagggtcaag	tgtaaaacagcacgtcatcctt
Podocin (NPHS2)	taccaaatcctccggcttagg	tttggctcttccaggaagcaga
SLC5A12	ttgtgggcttcttaacggttc	cgcctgagaggatctacatca
SLC9A3	ttgaggaggtccatgtcaacg	gcgccacgaaagattcaaaca
SLC17A3	aagaacgcacaagatatgcaagt	tgtaagacgagggctattccat
SLC22A7	actttcttcttcgccggtgt	attacatagctgacggaggctg
UMOD	actacgtctacaacctgacagc	tctatactgcactcctcacacg
VCAM1/CD106	cagtaaggcaggctgtaaaaga	tggagctggtagaccctcg

### Somatic mutagenesis in the kidney proximal tubule predisposes to the acquisition of driver mutations

**Fig. 5:**
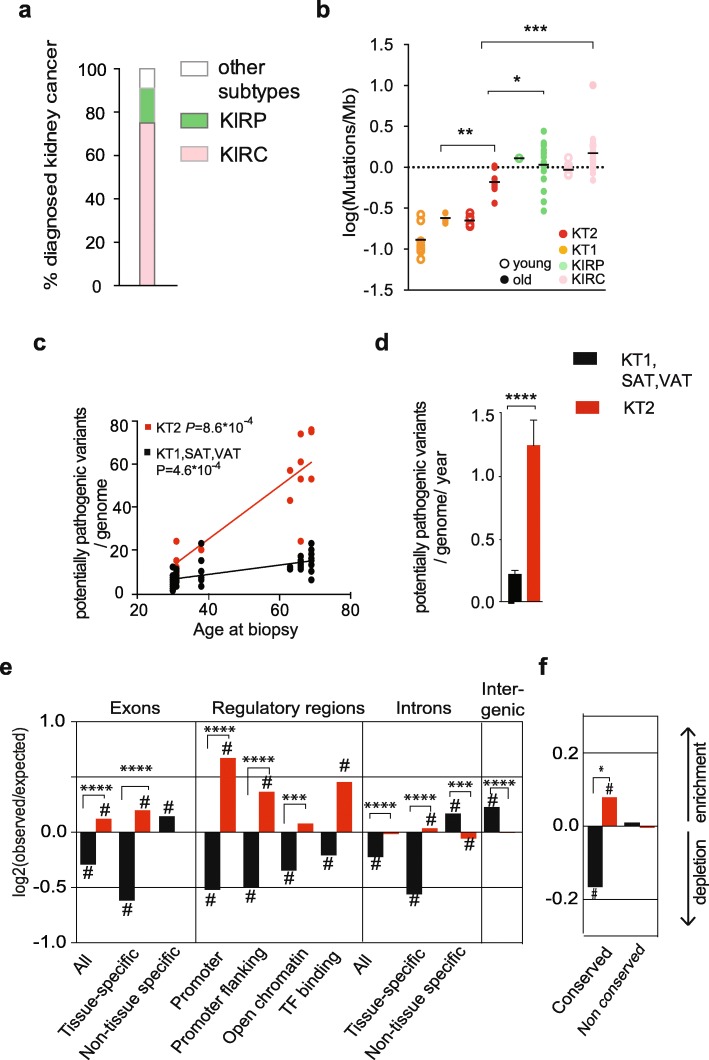
Kidney PT shows a unique somatic mutation pattern that confers high risk for tumor transformation. **a** Epidemiologic data showing the percentage of kidney tumors either derived from the proximal tubule, such as KIRC (clear cell renal cell carcinoma) and KIRP (papillary cell renal cell carcinoma), or from other kidney structures (other subtypes). **b** Somatic mutation burden in KT1, KT2, KIRP, and KIRC of either a younger (30–40) or older (60–70) age range. Significance among older groups was measured by one-way ANOVA. **c**, **d** Linear fit with age (**c**) and yearly increase (**d**) of potentially pathogenic variants in KT2 vs KT1-SAT-VAT clones. Potentially pathogenic variants are defined as follows: all variants were annotated with CADD (Combined Annotation Dependent Depletion; https://cadd.gs.washington.edu/). SNVs and InDels predicted to affect the coding sequence (presenting CADD score > 15) were selected and subsequently filtered on expression data in order to select only variants affecting a gene actually expressed in the tissue of origin of the clone. Tissue-specific and non-tissue-specific genes correspond to the expressed and non-expressed genes in the corresponding tissue according to the Human Protein Atlas (http://proteinatlas.com). Adjusted *P* values of the linear fit are calculated with the linear mixed model (**c)** or two-sided *t* test (**d**). **e** Enrichment (upward bars) or depletion (downward bars) of somatic mutations in indicated genomic features. The log2 ratio of the number of observed and expected point mutations indicates the effect size of the enrichment or depletion in each region. Log2 = 0 corresponds to a number of observed mutations equal to the number expected by random distribution. **f** Enrichment (upward bars) or depletion (downward bars) of somatic mutations in conserved and non-conserved regions of the genome. ^#^*P* < 0.05, one-sided binomial test. ***P < 0.001, ****P < 0.0001 two-sided *t* test of log2 ratios for either KT2 or KT1-SAT-VAT in specified genomic regions. EP epidermis, KT1 kidney tubule 1, KT2 kidney tubule 2, SAT subcutaneous fat, VAT visceral fat

Tumors derived from the PT (KIRC and KIRP) constitute the vast majority of tumors diagnosed in the kidney (Fig. 5a) [40], supporting the hypothesis that somatic mutagenesis in the PT favors tumorigenic transformation. Since KT2 are non-cancer clones from the PT of healthy kidneys, we studied these cells as a model of mutagenesis in the PT, prior to cancer initiation.

First, we confirmed that KT2 were not cancer clones at the moment of isolation from the tissue by analyzing the possible presence of the genetic lesions that commonly drive cancer initiation in KIRC and KIRP [41]. KT2 showed lower mutation burden compared to KIRC and KIRP (Fig. 5b) and did not display the typical kidney cancer genetic lesions, nor mutations in *TP53*, a tumor suppressor often mutated in pre-cancer clones in human tissues [7, 8, 10] (Additional file 1: Table S5). Yet, the mutation burden in cells from 63- to 69-year-old donors was higher in KT2 compared to other kidney cells (KT1; Fig. 5b) and the specific mode of somatic mutation accumulation in the PT could facilitate the acquisition of driver mutations and ultimately promote tumor initiation.

Kidney tumors are very rare at 30 years of age, but the incidence increases constantly and peaks in the 8th decade of life [40]. To model driver mutations, we selected the somatic mutations predicted to have a functional effect on a gene that is actually expressed in the tissue of origin. We defined these variants as potentially pathogenic mutations and determined their age-related increase (Fig. 5e, f). KT2 cells acquired higher numbers of potentially pathogenic mutations compared to other cell types from the same donors (KT1-SAT-VAT, Fig. 5e, f). The yearly increase was 5.7-fold higher in KT2 compared to KT1-SAT-VAT (Fig. 5f). From these data, we estimate that each PT cell accumulates an average of 86.5 potentially pathogenic mutations by the age of 70. A higher rate of accumulation of potentially pathogenic mutations makes the acquisition of cancer driver mutations in PT cells a more likely event compared to other cell types. These data are in agreement with the overall higher somatic mutation burden in KT2 (Fig. 2c-f). However, we also noticed that the mutation load in introns and exons of transcribed genes was higher than expected by random distribution and higher compared to non-expressed introns and exons (Fig. 5d). Conversely, the other cell types from the same donors (KT1-SAT-VAT, Fig. 5 d and Additional file 1: Figure S6) showed mutation depletion in these regions, in agreement with previous reports [11, 12]. Similarly, conserved regions were protected from mutations in KT1-SAT-VAT and enriched in KT2 (Fig. 5e). Finally, KT2 showed a particularly strong enrichment of mutations in regulatory regions (Fig. 5d). Overall, our somatic mutation analysis of non-cancer cells points to substantial differences in the genomic distribution of mutations depending on the cell of origin. These differences make specific cell types more vulnerable to the acquisition of mutations that affect the function of important genes, and this feature correlates with increased chances of a transition to cancer.

### Different efficiency of DNA repair in cells exposed to basal mutagenesis or additional mutagens

The regional pattern of distribution of mutations across the genome is shaped not only by mutagen exposure, but also by DNA repair. In fact, transcribed DNA is generally depleted of mutations due to the activity of the transcription-coupled NER (TC-NER) [25, 42]. In addition, mismatch repair (MMR) more efficiently protects from mutations the early-replicating and H3K36me3-rich DNA [21, 43]. Transcribed genes are usually located in early-replicating and H3K36me3-rich chromatin and benefit of both high TC-NER and MMR activities. Specific alterations in the pattern of regional differences of mutation accumulation are signs of TC-NER and MMR defects [21, 25, 42–44]. Therefore, we analyzed these patterns in our catalogue of healthy genomes.

**Fig. 6:**
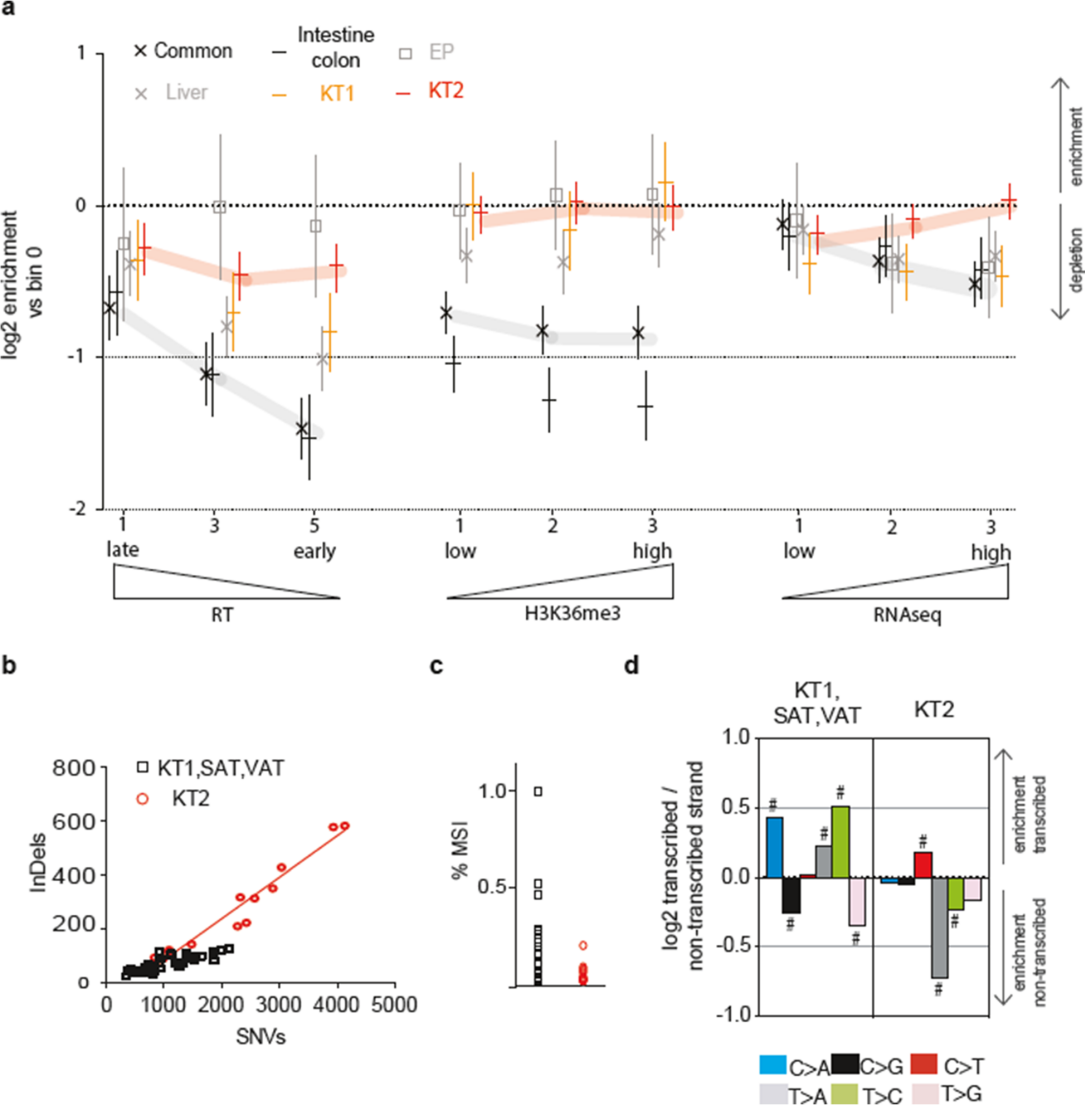
Mutation enrichment in specific genomic regions provides information on DNA repair efficiency and mutagen exposure in different cell types. **a** Enrichment/depletion of mutations in specific genomic regions. The genomes were divided in multiple sectors (bins) according to decreasing DNA replication time (RT, bins 0 to 5. For clarity, only bins 1, 3, and 5 are shown), increasing abundance of the histone mark H3K36me3 (bins 0–3), and increasing transcriptional levels (RNA-seq, bins 0–3). The relative abundance of mutations in each bin vs bin 0 for every tissue (EP, liver, KT1, KT2) or tissue group (common progenitors: SAT, VAT, SkM, blood; intestine-colon) is estimated as the coefficient in negative binomial regression (expressed as log_2_), where error bars show its 95% C.I. **b** Linear regression of SNVs and InDels per genome in the KT2 vs KT1-SAT-VAT group. **c** Percentage of sites subjected to microsatellite instability (MSI) in each genome of either the KT2 or the KT1-SAT-VAT group. **d** Enrichment of the six classes of substitution types in either transcribed or non-transcribed strand of genes. The log2 ratio of the number of observed and expected point mutations indicates the effect size of the enrichment in the transcribed (upper) or non-transcribed (lower) strand. ^#^*P* < 0.05, one-sided binomial test

Figure 6a shows the specific contribution of early/late DNA replication timing (RT), abundance of H3K36me3 marks, and transcription levels to the enrichment/depletion of mutations in different cell types. The group of common progenitors, including SAT, VAT, SkM, and blood, but not KT1, showed the expected depletion of mutations with earlier RT, higher H3K36me3 abundance and higher transcription levels (Fig. 6a and Additional file 1: Figure S7a-b). This pattern indicates that the basal mutagenesis is actively counteracted by MMR and/or TC-NER. However, EP, KT2, KT1, liver, SkM-long, and SkinFB deviated from the pattern seen for common progenitors and showed a loss of association of mutation rates with RT and H3K36me3 (Fig. 6a and Additional file 1: Figure S7c).

KT2 showed a severely affected RT and H3K36me3 pattern (Fig. 6a), thus suggesting that many mutations escaped MMR activity. While an increased proportion of InDels compared to SNVs in KT2 genomes was consistent with MMR defects (Fig. 6b), no evidence of a classical form of microsatellite instability (MSI) was detectable (Fig. 6c). These data suggest that some form of MMR is likely operative in these cells. Interestingly, KT2 were the only cell types displaying higher amounts of mutations in highly transcribed regions, while in all other cell types transcription protected from mutations (Fig. 6a, right). This suggests that a transcription-coupled mutagenic process [45] may be active in KT2 cells, supported by a striking, altered pattern of transcription-strand asymmetry of the different substitution types (Fig. 6d).

Overall, these results indicate a mechanism in cells that are exposed only to basal mutagenesis for sparing early-replicating-, H3K36me3-rich and highly transcribed regions from mutations. This occurs in diverse tissue types and is consistent with previous evidence of a more efficient activity of MMR and NER pathways directed towards active chromatin [22, 42]. In cells putatively exposed to a mutagen (EP, KT2, KT1, liver, SkM-long, and SkinFB), the altered, mutation-depletion pattern suggests that NER- and/or MMR-mediated protection is not as effective. KT2 cells show a unique pattern of mutation distribution that explains the higher mutation rate in transcribed genes (Fig. 5e).

### Aging affects the efficiency of MMR and NER

**Fig. 7:**
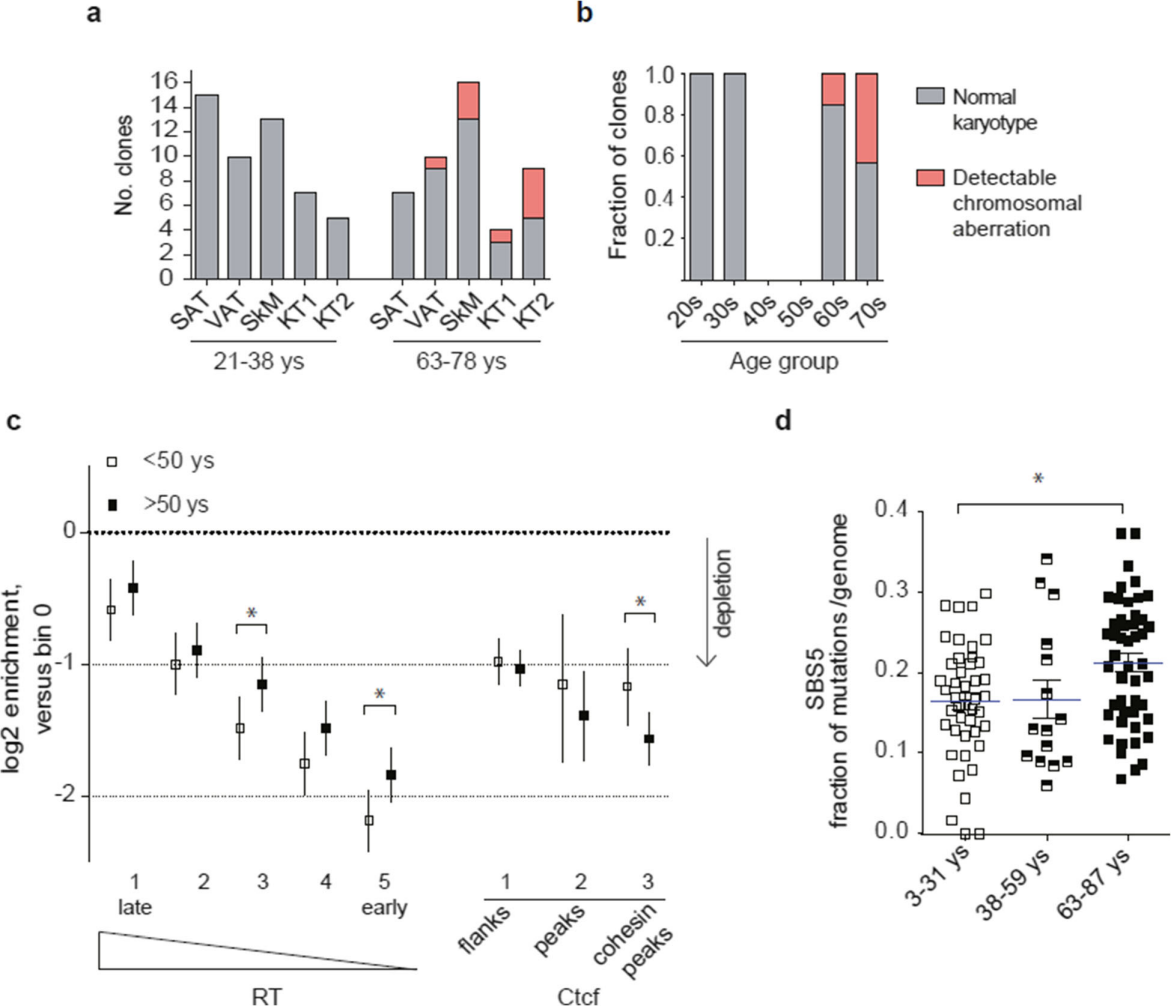
Genomic instability and weakening of DNA repair with aging. **a** Number of clones showing large chromosomal aberrations per tissue and age group. Young 21–38, old 63–78. **b** Fraction of genomes showing large chromosomal aberrations in the samples analyzed in **a**, but divided in tighter age groups (10 year-span). **c** Enrichment/depletion of mutations according to DNA replication timing (RT) while controlling for CTCF binding sites in either younger (< 50 years old, *N* = 52) or older (> 50 years, *N* = 54) genomes from the tissues not showing signs of exposure to external mutagens (SkM, SAT, VAT, intestine, and colon, according to the analyses in Figs. 3 and 6). Enrichments are coefficients from negative binomial regression (as log2), and error bars are their 95% C.I. Significance of young-vs-old differences was tested via a *Z*-test on the interaction term between age and replication time bin **d**. Fraction of SBS5 mutations per genome in different age groups of SkM, SAT, VAT, blood, intestine, and colon cells. *P < 0.05, one-way ANOVA and multiple comparison tests

Finally, we focused on non-tissue-specific effects of aging. Chromosomal instability is known to increase with age in normal tissues [2, 46]. Sequencing data from the 69 genomes from KT, SAT, VAT, and EP samples from 6 healthy kidney donors and 29 SkM progenitor genomes from 7 healthy donors from [11] were used to detect large chromosomal aberrations (Additional file 1: Table S6). These aberrations were recovered in three different tissues, i.e., skeletal muscle, VAT, and kidney tubules (both KT1 and KT2 cell types), but only in association with aging (Fig. 7a, b), supporting a general age-related increase of chromosomal instability.

The number of SNVs and InDels per genome also increased in all surveyed tissues with aging (Fig. 2c, d). To explore whether an age-related decline in DNA repair could contribute to somatic mutation accumulation, we selected cell types showing the more effective MMR and NER activities (Fig. 6a and Additional file 1: Figure S7a-c) and analyzed differences in mutation distribution and spectra in different age groups. Older genomes showed a weakened association of mutations with RT compared to younger ones, indicating a partial loss of MMR activity (Fig. 7c and Additional file 1: Figure S8a). The effect size of this defect was approximately one third of that observed in tumors with known MMR loss (MSI-H) (Additional file 1: Figure S8b), suggesting that aged, healthy cells acquire an early-stage mutator phenotype. MSI tumors were also found to lack mutations in binding sites for CTCF and Cohesin, in agreement with the requirement of a functional MMR to produce mutations at these sites [47]. Relative amount of mutations at CTCF/Cohesin peaks was lower in old vs young genomes. This result constitutes a further proof in support of a partial defect of MMR activity in old cells.

To investigate if defects extend to other pathways, we analyzed the age-related increase of SBS5, known to be associated with NER inactivation [19]. Results show that the fraction of SBS5 mutations per genome increases with age progression (Fig. 7d). This age-related expansion was specific for SBS5 and not detectable for the other ubiquitous signatures SBS1 and SBS3/8 (Additional file 1: Table S3b); this supports the hypothesis that NER weakens with advancing age. In summary, evidence demonstrates the decline of both MMR and NER in the genome of healthy cells as they age. This phenomenon is conserved across different tissues and occurs in cells that did not show genomic evidence of exposure to extrinsic mutagens.

## Discussion

We present here the basis of a somatic mutation atlas that can systematically guide the identification of cancer-prone cell types and high-risk somatic mutation processes. This collection exclusively includes whole genome data and high-confidence somatic variants obtained from single human cells, clonally expanded in vitro*.* Our newly generated data from the kidney, epidermis, subcutaneous fat, and visceral fat are based on samples derived from multiple tissues from the same individual. This strategy provides the advantage of a reliable comparison of tissue-specific differences, excluding the variability derived from different genetic backgrounds and environmental exposure. Newly generated data are complemented and compared with publicly available data sets from either healthy donors [11–13, 15] or tissue-matched cancer samples from TCGA and ICGC, for a final catalogue of 353 genomes and 12 different healthy cell types.

The comparison of somatic mutation landscapes in different cell types enables the identification of cells more susceptible to somatic mutagenesis and consequent cancer initiation [3]. This knowledge is expected to promote significant therapeutic advantages, including more targeted and efficient means of cancer prevention [3]. A major result of our analysis is recognizing that mutagen exposure can be very different even within the same tissue, and this correlates with different susceptibility to cancer initiation. It is possible that analysis of great numbers of genomes will uncover the concomitant presence of multiple cell subsets showing distinct mutation spectra in most tissues. We provide here the proof of principle by characterizing two populations of proliferating cells residing in the kidney tubule, one likely derived from de-differentiated epithelial cells of the proximal tubule (PT) and the other presenting features of undifferentiated kidney tubule progenitors. The somatic mutation spectrum of PT-derived cells presents unique characteristics that could not be identified in any other kidney or non-kidney cell. PT-derived cells showed the highest yearly increase of mutations among the cell types analyzed and a high incidence of the signature SBS40. The only samples that showed similar levels of SBS40 were kidney cancers derived from the PT, namely the clear cell and papillary cell RCCs (KIRC and KIRP, respectively). This analogy suggests that there is a specific process ongoing in the kidney PT and this process underlies the signature SBS40. Unfortunately, the etiology of this signature has not yet been determined. However, the extensive screening of cancer samples that identified SBS40 highlighted its predominance in kidney cancer [18]. Nonetheless, high levels of this signature have also been found in sporadic cases of tumors derived from multiple tissues, including the lung, skin, esophagus, bladder, head, intestine, stomach, liver, and ovary carcinoma, thus supporting the hypothesis that the mutagen causing SBS40 is more common, but not exclusively present in the kidney [18]. PT cells also displayed a unique distribution of mutations across the genome. The regions that are commonly spared from mutations as a consequence of more intense MMR and NER activity [21, 25, 42, 43] presented equal or higher mutation load compared to the rest of the genome. In particular, highly transcribed genes were enriched of mutations and the distribution of the different substitution types on the transcribed and non-transcribed strand was altered. These data indicate not only inefficient DNA repair, but also the presence of a mutagenic process that is more active on transcribed DNA. An important consequence of this unique mutation pattern was a mutation enrichment in functional genes and an age-related accumulation of high-risk mutations that was 5.7-fold faster in PT cells, compared to other cells from the same individuals. We estimated the presence of 86 mutations altering the protein sequence of expressed genes in every PT cell of 70-year-old individuals. Absolute numbers and other estimates of age-related increase of mutations presented in this work will be more accurate when a larger number of cells, distributed along the whole spectrum of ages, are analyzed. In addition, our numbers are certainly an underestimation, since our somatic mutation detection has a false-negative rate of 0.41 and does not allow the detection of all the variants present in a clone. However, our estimates support a strong acceleration in the appearance of pathogenic mutations in the genome of PT-derived cells. Mutations in the non-coding portion of the genome are also expected to affect the function of the cell, and we detected an enrichment of mutations in regulatory regions which is expected to significantly impact on overall gene expression. The high-risk somatic mutation landscape that we describe in PT cells predicts an elevated rate of tumorigenic transformation in this portion of the nephron. In agreement, somatic mutagenesis is recognized as a major tumorigenic mechanism in the kidney [41, 48, 49] and the PT-derived tumors KIRC and KIRP constitute up to 95% of all cancers diagnosed in this organ [36, 40]. Therefore, our analysis points to PT cells as a cell type at particularly high risk of tumor transformation. A clear understanding of the underlying mutational mechanisms can be exploited to slow down mutation accumulation and kidney cancer incidence.

The comparison of mutational profiles observed in healthy cells with the landscape of mutations observed after in vitro exposure to common mutagens [33] provides interesting hypotheses about the mutagens active in the kidney PT. The genomic modifications observed in healthy PT cells or tumors derived from the PT were similar to those induced by formaldehyde and alkylating agents [33]. Alkylating agents used in [33] are chemotherapeutic drugs, such as 1,2-dimethylhydrazine and diethyl sulfate. The healthy kidney donors from which cells were isolated were never treated with those agents nor exposed to formaldehyde. Therefore, we hypothesize that the mutation spectrum might be due to the action of endogenously produced compounds that interact with the DNA in a similar way as the synthetic drugs [35]. Indeed, the epithelial layer of the kidney PT presents a complex chemical environment that is the consequence of ongoing physiological activities, such as ammonia production and excretion, amino acid reabsorption and modification, and transformation and excretion of xenobiotics [50]. Further analyses might support a link between the presence of these compounds in the kidney PT and enhanced mutagenesis in this specialized epithelium.

The kidney PT is an example of particularly high and specific mutagen exposure. However, our analysis also found cell types that are broadly protected from mutagens and constitute a model of minimal or “basal” mutagenesis. These cells are progenitors from multiple, unrelated tissues, namely skeletal muscle, kidney tubules, blood, and both subcutaneous and visceral fat. Unexpectedly, these different cell types present a somatic mutation profile that is strikingly similar. This finding is in contrast to the hypothesis of a tissue-specific mutation profile consequent to different activities and mutagen exposure in each tissue [2, 17]. The absence of tissue-specific mutagen exposure constitutes a simple way to explain how different cell types can share the same mutation profile. In this perspective, mutations observed in skeletal muscle, kidney tubules, blood, and fat progenitors are necessarily the consequence of common cellular activities, such as “house-keeping” activities. In support of this hypothesis, this group of cells, which we named “common progenitors,” displays the lowest age-related increase of mutations among the cells analyzed. In addition, the signatures characterizing the common profile are found ubiquitously, but most cell types accumulate other tissue-specific mutations in addition to the common profile.

The lack of exposure to tissue-specific mutagens in the common progenitors is not surprising since tissues, like the skeletal muscle and blood, have stem/progenitor cells that reside in a protected microenvironment and are shielded from damage [51]. Somatic mutation profiles are a record of the cell lineage and activities during an individual’s lifetime. Therefore, somatic mutation data can be used to address unsolved questions about stem cell hierarchy and tissue architecture [13, 52]. In the kidney, the existence of resident stem cells is controversial and the presence of a potential, protective niche is debatable [53]. Presently, the regeneration of damaged KTs appears to be mediated by (1) resident progenitors [39] and (2) tubule-epithelial cells that lose their differentiation and reacquire proliferative capacities [38]. Our analysis of the somatic mutation landscape supports both types of progenitors. Cells with in vitro proliferative capacities derived from human KTs showed either a mutation profile similar to the resident progenitors of fat and SkM (consistent with a resident KT stem cell) or a profile similar to PT-derived tumors and signs of cellular damage at both DNA and RNA level (consistent with a de-differentiated cell). The two populations do not seem completely separated. In agreement, we found a genome from a 38-year-old individual that showed an intermediate mutational and expression profile. The population of uncommitted KT progenitors also showed signs of mutagen exposure when we explored the distribution of mutations. This is consistent with their location in an environment that is not completely protected. We hypothesize that they reside in the PT, but are not part of the epithelial layer. Finally, our analyses also explored potential differences between adipose tissue progenitors residing either in the subcutaneous or visceral fat. SAT and VAT are considered two different tissues and show important differences, especially concerning the morphological changes occurring with aging [29]. However, our somatic mutation data do not support specific differences in mutagen exposure in progenitor cells from the two different types of fat during aging.

The finding and characterization of an age-related process that most likely occurs in every cell throughout the human body is a major finding of this study. This phenomenon has been termed here as “basal mutagenesis.” Somatic mutation analysis in cancer genomes has identified two signatures that present clock-like features, i.e., inevitable increase in all cells as the human body ages [54]. These signatures are considered to be the products of core cellular processes, such as spontaneous deamination of methyl-cytosines (signature 1) and polymerase errors that escape the DNA repair system (signature 5) [17, 19, 20]. Results from our study expand the clock-like concept and define basal mutagenesis directly in non-cancer genomes from healthy, human tissues. Besides signatures SBS1 and 5, basal mutagenesis includes a signature that is similar but does not completely overlap with SBS3 and SBS8. In addition, we propose that SBS5 increases in a clock-like way in most cell types, but can also be enhanced by specific mutagenic processes, as observed in liver stem and kidney PT cells.

Our characterization of basal mutagenesis also includes the distribution of mutations in relation to specific, genomic features and the impact on DNA repair over time. Thanks to the comparison of older vs younger samples from multiple tissues, we are able to determine a loss of efficiency of MMR coupled with aging. In particular, the MMR-mediated protection of early-replicating DNA deteriorates with aging. We estimate that the effect size of this defect is one third of that observed in tumors with a complete MMR deficiency. These results show that the rate of somatic mutagenesis increases with aging especially in the gene-rich, early-replicating DNA, overall increasing the chances of acquiring cancer driver mutations. In addition, we found that samples from aged individuals were subjected to a relative expansion of mutations attributed to SBS5, a signature that is enhanced by another DNA repair pathway, NER. Overall, these findings suggest that the efficiency of DNA repair, in particular the MMR and NER pathways, is decreased in aged cells. These evidences point to the loss of DNA repair as an accelerating factor in cellular aging and open the door to innovations in pharmacology.

## Conclusions

We provide a comprehensive genome-wide analysis of somatic mutagenesis in human cells. Our model of basal mutagenesis offers an enhanced understanding of the unavoidable loss of genome integrity and the protective forces that counteract this process, including the stem-cell niche and DNA repair. The finding of cell-type-specific mutagen exposures and consequences on cell fate in the kidney are a proof of principle supporting the importance of understanding mutational processes active in healthy human cells to understand cancer. WGS data from single genomes constitute a precious tool for achieving the goal because they allow the analysis of the non-coding portion of the genome. Overall, our comprehensive classification of mutagenic processes introduces a novel perspective for clinical advancements in preventing cancer- and age-related diseases.

## Methods

### Clonal cultures from multi-organ biopsies from kidney donors

Human biopsies were obtained intra-operatively from healthy living kidney donors, according to Ethical Permit Dnr 2015/1115-31. From the explanted kidney of each donor, a needle biopsy from the kidney cortex and a piece of suprarenal fat were obtained. In addition, a piece of skin with annexed subcutaneous fat was obtained. Tissues were preserved in cold PBS and immediately processed for cell isolation.

### Isolation and clonal expansion of tubular progenitors from human kidney biopsies

Using a needle biopsy (1 mm diameter/10 mm height), 7–8 mg of tissue from the kidney cortex of the explanted kidney were obtained intra-operatively. The protocol for cell isolation and culturing was adapted from [55, 56]. Tissue was minced in tiny pieces with a scalpel. Around 1/5 of the biopsy was used for direct DNA/RNA extraction from whole kidney tissue. The rest was resuspended in medium and passed through tissue strainers with mesh sizes of 100 and 70 μm, thereby excluding glomeruli from the preparation. The tubular portion, which had passed through the cell strainers, was pelleted, then treated with 1× trypsin–EDTA for 5 min at 37 °C and gentle agitation, then mixed with medium and passed through a 40-μm strainer to obtain a single cell suspension. FACS sorting of CD133+ cells and single cell clonal expansion in 96-well plates was attempted (*n* = 4 biopsies) using the clone AC133 antibody (Milteny biotec, Bergisch Gladbach, Germany), but was unsuccessful. To obtain clone growth, single cell suspensions were directly plated in 6–8 wells of 6-well microtiters at 37 °C and 5% CO_2_. Culture dishes were fibronectin coated (Sigma-Aldrich) and culture medium was EBM + EGM-2 MV BulletKit (Lonza, Basel, Switzerland). Twenty-four hours after plating, the medium was changed. First, the plating medium was collected and re-plated in a new 6-well microtiter to allow further attachment of kidney progenitors. One week after plating, 1–20 colonies per/well were distinguishable. Colonies with round shape and tight cell-cell contacts were considered for further culture, while scattered cells were discarded (Additional file 1: Figure S1b). When reaching ≈ 1000 cells, colonies were detached with trypsin, manually picked, and moved to new fibronectin coated 6-well microtiters, one colony per well. The whole procedure was performed under stereomicroscope inspection. Colonies were grown until confluence and used for DNA extraction. Clones that reached confluence within 1 week were moved to 10-cm-diameter petri dishes. Mean time in culture was 27.9 ± 0.8 days (*n* = 26 clones from 6 biopsies).

To assess the effectiveness of the culturing strategy, a selection of clones was subjected to FACS analysis of tubular progenitor markers [39] and qPCR analysis for markers of different kidney cell types. One hundred thousand cells per clone were stained for the kidney tubule progenitor markers CD133 (clone AC133) and CD24 (clone 32D12, both from Milteny biotec, Bergisch Gladbach, Germany) and analyzed with FACS (FACSCalibur™ - BD Biosciences). The percent of double positive cells was calculated by comparison with cells from the same clone stained with matching control IgGs (Milteny biotec) (see also Additional file 1: Figure S1c). A subset of sequenced and non-sequenced clones was also tested for the expression of transcripts considered markers of different cell types present in the kidney (see Additional file 1: Figure S1e and the section “RNA extraction and qPCR” in the “Methods” section). FACS and qPCR analyses of expression of kidney cell markers in KT clones were performed after 3–5 weeks in culture. To avoid loss of cells from clones meant for sequencing, only selected sequenced clones were inspected for the expression of kidney markers: P4903_104; P4903_117, P4903_118, P4903_119, P4903_131, P4903_132, tested by FACS; P4206_106; P4206_107; P4206_122; P4903_102, tested by qPCR; and P4903_128 and P4903_131, tested by both FACS and qPCR. The analyses were extended to clones not used for the sequencing (non-sequenced clones). These clones either came from a test biopsy (*n* = 7, female individual, age 57) or were selected among non-sequenced clones from individuals KD10 (*n* = 3), KD11 (*n* = 4), and KD12 (*n* = 11).

### Clonal expansion of fat progenitors from human biopsies

One to ten grams of abdominal subcutaneous (external to the fascia superficialis) and visceral (peri-renal) fat were obtained from kidney donors undergoing surgery according to Ethical Permit Dnr 2015/1115-31. Part of the tissue was frozen for direct DNA/RNA extraction. The rest was accurately rinsed, cleaned of visible vessels, and minced with a scalpel. Tissue was placed in 30–50 ml of Hank’s balanced salt solution (HBSS) containing 1 mg/ml collagenase (Collagenase A, Roche, Basel, Switzerland) in a 37 °C shaking incubator until complete digestion (30–40 min). To separate the stromal vascular fraction (SVF) from mature adipocytes, the digested tissue was centrifuged at 500*g* for 10 min and the supernatant discarded. The SVF pellet was resuspended in 1 ml of erythrocyte lysis buffer (RBC lysis solution, Qiagen) at room temperature for 5 min. To stop the lysis, cells were pelleted by centrifugation at 500*g* for 5 min and supernatant discarded. SVF was resuspended in medium and filtered through a 40-μm strainer, then plated in a 10-cm-diameter culture dish with low-serum plating medium (Dulbecco’s modified Eagle’s medium (DMEM)/Ham’s F-12, Life Technologies that contained 0.5% bovine serum). After 12 h in a 37 °C and 5% CO_2_ incubator, non-adherent cells were carefully washed away and adherent pre-adipocytes were detached by 3–5 min of trypsinization. Cells were rinsed and stained for the hematopoietic marker CD45-APC (clone HI30, BD Biosciences, USA) and the endothelial marker CD31-PE (clone L133.1, BD Biosciences). CD45^neg^ CD31^neg^ fat progenitors were FACS sorted using a BD FACSAria™ Mu cell sorter (BD Biosciences) (see Additional file 1: Figure S1f) and single cell plated in uncoated 96-well culture plates, one plate/biopsy. Additional cells were sorted in 6-well plates as a population of 10,000–30,000 pre-adipocytes, 1 well/biopsy, and grown for 1 week before freezing. The plating medium (DMEM F12 10% FBS) of single cell cultures was changed every 2 days. The number of colonies was scored at 2 weeks after plating. At confluence (around 3 weeks), cells were trypsinized and moved to 24-well plates. Depending on the cell confluency, the colonies were then moved to 6-multiwell plates. After an average of 46.2 ± 1.3 and 48.0 ± 1.5 days in culture for subcutaneous and visceral fat, respectively, the colonies were confluent and used for DNA extraction.

### Clonal expansion of epithelial progenitors from human biopsies

Skin biopsies from the lower abdomen were obtained from kidney donors undergoing surgery. The tissue was placed in cold HBSS without Ca^2+^and Mg^2+^(Life Technologies) containing antibiotics and antimycotics (Anti-anti, Gibco, Life Technologies) and kept at 4 °C for 4–6 h. Subcutaneous fat and loose connective tissues (hypodermis) were carefully removed. The tissue was flattened and cut into strips about 3–4 mm wide. The pieces were placed with the dermal side down in a dish containing HBSS with antibiotics and dispase (Corning, USA) and kept at 4 °C overnight. The digested epidermis was peeled from the dermal side, minced, and trypsinized with TrypLE Select (Gibco, Life Technologies) at 37 °C for 30–40 min. The digested tissue was passed through a 70-μm mesh filter, collected in a new tube containing medium and centrifuged. Pellet was resuspended in EpiLife medium, filtered through a 40-μm strainer and plated in 4 wells of a 6-well multiwell coated with collagen (5 μg/cm^2^ of Collagen I bovine protein, Gibco, following the “thin coating procedure”). Growth medium was EpiLife medium (Gibco, Life Technologies), no serum. The procedure did not produce any colonies for individuals KD05, KD09, KD10, KD11, and KD12. The culture of the epidermis from individual KD06 produced 2 colonies. Colonies of small, tight, and fast proliferating cells were visible on the extremities of the dish starting from 2 weeks after plating. When reaching ≈ 1000 cells, colonies were detached with trypsin, manually picked, and moved to new collagen-coated 6-well microtiters, one colony per well. The whole procedure was performed under stereomicroscope inspection. The cells tended to differentiate into mature large keratinocytes (see the picture in Additional file 1: Figure S1a), but a portion of cells kept small size and very high proliferative capacity for multiple passages. DNA was extracted 34 days after initial plating.

### DNA extraction

DNA was extracted from the confluent wells of the 6-multiwell plate using the Gentra Puregen Kit, Qiagen. DNA was extracted from tissue biopsies using the Gentra Puregen Kit, supplemented with a lysis buffer containing Proteinase K as recommended by the supplier. DNA was extracted from 3 ml of total blood that was collected in EDTA as recommended by the instructions of the Gentra Puregen Blood Kit.

### Sequencing

The library preparation and sequencing were carried out at NGI Sweden, Science for Life Laboratories, Stockholm, following standard methods. For cell clones, the library preparation was performed by a semiautomatic NeoPrep station using the Illumina TruSeq Nano Kit (350 bp average insert size) and 25 ng of DNA as starting material. The libraries of the bulk blood samples were prepared with Illumina TruSeq PCR-free library preparations (350 bp average insert size). Sequencing was performed on Illumina HiSeq X, PE 2 × 150 bp.

### Somatic variant calling

Raw reads were aligned to the human reference genome (GRCh37/hg19 assembly version), using bwa mem 0.7.12 [57]. Alignments were sorted and indexed using samtools 0.1.19 [58]. Alignment quality control statistics were gathered using qualimap v2.2 [59]. The raw alignments were then processed following the GATK best practice [60] with version 3.3 of the GATK software suite. Alignments were realigned around InDels using GATK RealignerTargetCreator and IndelRealigner, duplicates were marked using Picard MarkDuplicates 1.120, and base quality scores were recalibrated using GATK BaseRecalibrator. Finally, genomic VCF files were created using the GATK HaplotypeCaller 3.3. Reference files from the GATK 2.8 resource bundle were used. All above steps were coordinated using Piper v1.4.0 (www.github.com/NationalGenomicsInfrastructure/piper).

Somatic variants were defined as heterozygous in the single cell clone and either absent or very rare in an unrelated tissue (blood), sequenced as a bulk. To identify somatic variants, a specific pipeline was developed. For each clone, variants were initially called with HaplotypeCaller (GATK) [61], MuTect2 (GATK 3.5.0), and FermiKit version r178 [62]. The union of these three sets of variants was subjected to further filtering steps in order to exclude (1) sequencing artifacts, (2) germline variants (detected both in the clone and blood bulk), and (3) variants that occurred during the in vitro culture of the clone (found only in a subset of cells of the clone, therefore showing low AF). To this aim, the AF of each variant was derived from the .bam files and matched to the relative blood bulk sequencing. Somatic variants were defined as follows: the read fraction supporting the alternative allele was comprised between 0.4 and 0.6 in the clone sequence, a minimum of 3 reads supported the variant, the read fraction in the blood was low (alternative < 0.1), and the coverage in both the clone and blood was at least 15X. Chromosomes X and Y were excluded from the analyses (however, variants recovered on the X chromosomes of female donors can be found in Additional file 3). Additional quality filters were applied as follows: the reads supporting the variants were on both strands, the maximum coverage was 1000X, and the variants that were located in problematic regions [63, 64] were removed. Variants common to more than one sample were considered artifacts and removed. Variant validation was performed to ensure that our lists of somatic mutations only contained somatic variants that were present in the cell before in vitro culturing (see the section “Variant validation” in the “Methods” section). Comparison of variants recovered in DNA from a clone derived from the same ancestor cell, but cultured in 2 different wells and independently sequenced, shows high validation rate (99 and 97% for SNVs and InDels, respectively, Additional file 1: Table S1e) and supports low levels of culture-induced variants in our lists. However, we cannot exclude the presence of non-neutral, positively selected variants that might have occurred in vitro*.* Variants were annotated using the Ensembl Variant Effector Predictor from [65]. Frequency of detected somatic SNVs in the Swedish population (germline variants) was annotated in Additional file 2 and Additional file 3 using SweGen [66] version 20180409.

### Variant validation

The variant validation was performed on a technical replicate of WGS. Two clones derived from the same ancestor cell (P4206_128 and P4206_130) were independently grown in culture. The DNA was extracted and sequenced independently, but clone P4206_130 was not included in the study. Variants were called in clones P4206_128 (discovery set) according to our somatic variant calling pipeline. Called variants that had a minimum coverage of 10x in both the discovery and the validation sets were used for the validation. In total, 870 SNVs and 71 InDels were tested. Variants were considered validated when at least 3 reads supporting the alternative alleles were present in the validation set. As a control for the background signal, we validated the variants in unrelated clones, e.g., clones derived from a different founder cell obtained from the same or a different biopsy. Additional validation and discussion of our somatic mutation calling strategy are available at [11].

### Microsatellite instability

Microsatellite instability was assessed using MSIsensor v.0.5 [67] where every cell clone and representative blood bulk were analyzed and the msi score calculated.

### Copy number variation

Copy number variation was detected in clonally expanded cells using Ascat [68]. Ascat detects allele-specific copy number variation in a tumor sample using Log R and B allele frequency (BAF) information at specific SNP loci in the tumor sample and a matched germline sample from the same individual. We used the loci of all bi-allelic SNPs in 1000 Genomes phase 3, release date 20130502 [69] with minor allele frequency > 0.3 to calculate Log R and BAF data in the clonally expanded cells and the matched blood samples. The software AlleleCount (https://github.com/cancerit/alleleCount) was used to generate the number of reads in the bam files supporting the two alleles of the SNPs. BAF and LogR was then calculated at all SNP loci according to:

$$ {\mathrm{BAF}}_i^c=\frac{CountsB_i^c}{CountsA_i^c+{CountsB}_i^c} $$

$$ {\mathrm{BAF}}_i^b=\frac{CountsB_i^b}{CountsA_i^b+{CountsB}_i^b} $$

$$ {\mathrm{LogR}}_i^c={\log}_2\frac{CountsA_i^c+{CountsB}_i^c}{CountsA_i^b+{CountsB}_i^b}-\mathrm{median}\ \left({\log}_2\frac{CountsA^c+{CountsB}^c}{CountsA^b+{CountsB}^b}\right) $$

$$ {\mathrm{Log}R}_i^b=0 $$

where *i* is a specific SNP locus, *c* is the clonally expanded sample, *b* is the blood sample, *CountsA* is the number of reads supporting one of the alleles of the SNP, and *CountsB* is the number of reads supporting the other allele of the SNPs.

Ascat was run with parameter gamma set to 1. We report only large copy number aberrations that were detectable by visual inspection of the ASPCF.png and ASCATprofile.png images generated by Ascat for each sample. Execution of Ascat and the generation of Log R and BAF was coordinated using Sarek release v2.1.0 [70].

### Meta-analysis

Newly generated and publicly available somatic SNVs from normal and cancer samples underwent a common filtering step to exclude variants from the repeat-masked hg19 genome assembly. In particular, we excluded regions with CRG Alignability-75 score [71] below the maximum (< 1.0) and additionally the UCSC Browser blacklisted regions (DAC and Duke) were excluded; this step retained 2393.43 Mb of the genome. Furthermore, we excluded from all analyses the regions with low genomic coverage in our data (< 15 reads in WGS of > 5% of the samples), retaining 2094.95 Mb of the hg19 genome for the final analysis.

### Mutational signature inference

Analysis of mutational signatures was performed as described in [21]. Briefly, the SNVs from the healthy samples and the tumor samples were analyzed jointly, where a NMF (non-negative matrix factorization) analysis was applied to matrices of mutation counts across the 96 mutational contexts, as customary (see, e.g., [16]). Upon repeated runs (*n* = 200) of the NMF procedure (function *nmf* in the *R* package *NMF*, using the default “Brunet” algorithm) on the bootstrap-resampled mutation count data, the 200 NMF results were clustered using k-medoids algorithm (function *pam* in R package *cluster*) to obtain the final set of mutational signatures and their contributions (exposures) in every sample. The signature profiles obtained from this NMF analysis were compared using cosine similarity to the known mutational signatures (http://cancer.sanger.ac.uk/cosmic/signatures and [18]).

### Genomic distribution of mutations

Analysis of enrichment or depletion of mutations in exons, introns, regulatory, and conserved regions was carried on using the R package *MutationalPatterns* [72]. Tissue-specific genes were obtained from the Human Protein Atlas (http://proteinatlas.com). The genes that had the annotation “elevated in …,” “expressed in all,” and “mixed expression pattern” were considered tissue-specific gene for that tissue. To define the conserved regions, PhastConsElements46way data was used and downloaded from http://hgdownload.cse.ucsc.edu/goldenpath/hg19/phastCons46way/.

The association of mutation enrichment/depletion with specific genomic features was performed as described in [21, 44]. In brief, regression analysis was performed to examine the relationship between the mutations and the covariates (replication timing, H3K36me3, transcriptional levels, CTCF motif) individually while controlled for others. The replication timing (RT) data was obtained from the ENCODE project (RepliSeq) and divided into six bins ranging from latest replicating (bin 0) to earliest replicating (bin 5); values are averages over eight diverse cell types (source file names in the form “wgEncodeUwRepliSeq_____WaveSignalRep1.bigWig” where the gap contains cell line names: Helas3, Hepg2, Huvec, Nhek, Bj, Imr90, Mcf7, Sknsh). The RNA-seq levels and H3K36me3 histone mark were collected from Roadmap Epigenomics project and averaged over eight diverse cell types (for H3K36me3: E017 LNG.IMR90, E114 A549, E117 CRVX.HELAS3.CNCR, E118 LIV.HEPG2.CNCR, E119 BRST.HMEC, E127 SKIN.NHEK, E125 BRN.NHA, E122 VAS.HUVEC; for RNA-seq, these same cell types except that we substituted E096 and E071 for E017 and E125 because of data availability). The RNA-seq was divided into four bins where non-expressed regions were in bin 0 and expressed regions were in bins 1 (low expression) to 3 (high expression). The H3K36me3 was divided into four bins, with bin 0 as absent from H3K36me3 (fold-enrichment versus ChIP-seq “input” ≤1.0) and ranging up to bin 3 with the highest abundance.

### Predicted pathogenic variants

To obtain the number of potentially pathogenic mutations in each clone, SNVs and InDels were annotated with CADD (Combined Annotation Dependent Depletion) [73]. Mutations that obtained a PHRED score higher than 15 were selected and filtered on gene expression (obtained from Human Protein Atlas, as described in the section “Genomic distribution of mutations”). Variants with CADD score higher than 15, but no gene annotation were excluded, as well as variants affecting the sequence of a gene not expressed in the tissue of origin of the clone.

### RNA extraction and qPCR

RNA from KT clones was extracted from plated cells, previously snap-frozen in their tissue culture plates, using the RNeasy Mini kit (Qiagen), according to the manufacturer’s instructions. RNA from total kidney was obtained from a needle biopsy from a healthy kidney not included in the study (female, age 38) undergoing explant for kidney donation. The fresh biopsy was minced in tiny pieces, and around 1/5 of the material was snap-frozen for RNA extraction. The rest of the biopsy was used for KT progenitor culture. RNA from the biopsy was extracted using the RNeasy Mini kit (Qiagen) and homogenized with a syringe. RNA from all samples used in the qPCR analyses were extracted at the same time. cDNA synthesis was performed using random hexamers and SuperScript Reverse Transcriptase (Invitrogen). Quantitative RT-PCR was performed using either a TaqMan gene expression assay from Applied Biosystems (Podocalyxin, PDX, Hs00193638-m1) or SYBRgreen using the set of primers specified (Table 1).

### Statistical analyses

Unless otherwise indicated, the *P* values were calculated using either two-tailed distribution, two-sample unequal variance Student’s *t* tests (when comparing two groups), or one-way ANOVA with multiple comparison post hoc test. Significance was defined as *P* < 0.05 (*P < 0.05, **P < 0.005, ***P < 0.0005). The results are presented as the mean ± standard error of the mean (SEM). All calculations were performed using GraphPad Prism software. The linear fits between mutation numbers and age were obtained using a linear mixed-effects model where the dependent variable is the number of mutations or a given mutational signature, the fixed effect is age, and the random effect is the individual. Bonferroni correction was used to adjust for multiple testing. Analyses were performed in R. T-SNE analysis was performed using *tsne* package in R, and clustering showed in Fig. 2a was performed using *heatmap3* package in R.

## Supplementary information

**Additional file 1.** Supplementary figures (Figure S1-S8) and tables (Table S1-S6)

**Additional file 2.** Lists of somatic mutations detected on autosomes of 69 clones from 6 healthy donors and grouped per tissue

**Additional file 3.** Lists of somatic mutations detected on the X chromosome of female donors (KD05, KD06 and KD11). Not used in the analyses.

**Additional file 4.** Review history

## Data Availability

Sequencing data generated during the current study are not publicly available due to the European General Data Protection Regulation (GDPR) to protect patients’ privacy, but are available from the corresponding author on reasonable request. Aggregated lists of somatic variants recovered in all clones of all donors are available as Additional file 2 (autosomes) and Additional file 3 (X chromosomes of female donors). Lists of somatic variants used in the meta-analysis are either accessible from the original publication (listed in Additional file 1: Table S2) or available upon request from the GDC Data Portal (for the TCGA data set samples) and the ICGC Data Portal (sample IDs are listed in Additional file 1: Table S2).
