# Supplementary material for: Whole genome DNA sequencing provides an atlas of somatic mutagenesis in healthy human cells and identifies a tumor-prone cell type
Source: Genome Biol. 2019 Dec 18;20:285. doi: 10.1186/s13059-019-1892-z (PMC6918713; doi:10.1186/s13059-019-1892-z)
Supplement: Supplementary file 1 — Additional file 1. Supplementary figures (Figure S1-S8) and tables (Table S1-S6) [file 13059_2019_1892_MOESM1_ESM.docx]

**Additional file 1**

**Whole genome DNA sequencing of healthy human cells provides an atlas of somatic mutagenesis and identifies a tumor-prone cell type**

Irene Franco^1*^, Hafdis T. Helgadottir^1*^, Aldo Moggio^2^, Malin Larsson^3^, Peter Vrtačnik^1^, Anna Johansson^4^, Nina Norgren^5^, Pär Lundin^1,6^, David Mas Ponte^7^, Johan Nordström^8^, Torbjörn Lundgren^8^, Peter Stenvinkel^9^, Lars Wennberg^8^, Fran Supek^7,9^ and Maria Eriksson^1^

**Supplementary figures**

Fig. S1. Characterization of clonally expanded progenitors from human kidney tubules, fat and epidermis

Fig. S2. Non-negative matrix factorization and comparison of extracted signatures to COSMIC cancer signatures and PCAWG single base signatures (SBS)

Fig. S3. Relative contribution of extracted signatures to healthy tissues and tissue-matched tumors

Fig. S4. Comparisons of cancer and normal samples

Fig. S5. Comparison with mutation spectra determined by *in vitro* exposure to environmental agents

Fig. S6. Mutation enrichment in specific genomic regions in KT1, KT2, SAT, VAT samples and age-related differences

Fig. S7. Regional analysis of enrichment/depletion of mutations in different tissues

Fig. S8. Association of mutations with replication timing in young and old genomes of healthy samples and MMR-proficient (MSS) or deficient (MSI) tumors

**Supplementary Tables**

Table S1. Donors and samples

Table S2. Identity of samples used in the meta-analysis

Table S3. Differential contribution of specific signatures in differentb tissue-groups

Table S4. Age-related SBS signature changes in healthy samples

Table S5. Genes commonly mutated in KIRC/KIRP and SNVs, InDels and CNVs detected in 69 progenitor cells from healthy KT, SAT, VAT, EP

Table S6. Chromosomal aberrations detected in 69 progenitor cells from healthy KT, SAT, VAT, EP and 29 skeletal muscle progenitors

**Supplementary bibliography**

**Supplementary figures**

Fig. S1. Characterization of clonally expanded progenitors from human kidney tubules, fat and epidermis

Representative micrographs of single cell clones from human biopsies used in the study. Kidney tubule (KT, top left), epidermis (EP, top right), sub-cutaneous adipose tissue (SAT, bottom left) and visceral adipose tissue (VAT, bottom right) progenitors were expanded in culture for 3 to 6 weeks, then used for DNA extraction and sequencing. The presented pictures correspond to the final stages of the cell culture. The cell morphology was checked and used for selecting suitable clones for sequencing. **b.** Representative images and 5x magnifications (bottom) of colonies from KT cultures and criteria for selection of KT progenitor colonies on the base of morphology. Cultures from KT cell suspensions were inspected daily to follow the growth of distinct colonies. Ten to 15 days after plating, one colony per well was selected, detached and moved to a new plate. Only colonies with round shape and tight cell-cell contacts (left panels) were considered for further culture, while colonies composed of scattered cells (right panels) were discarded. Bars=50 μm **c.-e.** FACS and qPCR assessment of expression of kidney cell markers in KT clones after 3-5 weeks in culture. Due to the reduced amount of material obtained from the clonal culture, only a portion of the KT clones included in the somatic mutation analysis could also be tested for the expression of kidney markers. To extend the characterization, FACS and qPCR analyses were performed on clones not used for the sequencing (non sequenced clones), but cultured at the same time as the ones chosen for DNA extraction. Overall, all tested KT clones (n=20) expressed the markers of kidney progenitors CD24 and CD133, while fat clones used as negative controls were completely negative **(c.-d.).** In KT clones, CD24 was expressed by nearly all the cells within the clonal population, while the levels of CD133 were more variable (**c.**). Expression of the kidney progenitor marker *PAX2* was detectable in most KT clones at the RNA level **(e)**. Conversely, KT clones were always negative for markers of non-tubular cells, like *NEPH* and *PODO* (glomeruli), *PECAM* (endothelium), *ACTA2* (smooth muscle cells) and *COL1A1* (fibroblasts) **(e)**. A portion of a healthy kidney biopsy (Total kidney), a VAT clone and non-clonal populations of either embryonic stem cells (ESC bulk) or skin fibroblasts (SkinFb bulk) were included in the qPCR analysis as positive and negative controls **(e)**. Three clones from biopsy KD12 were tested by both FACS and qPCR **(c and e)**: the non-sequenced KT clone 3 (a representative clone that was excluded from sequencing on the base of morphological appearance of the cells, as described in **(a))** and two sequenced clones, P4903_128 and P4903_130. **f.** Representative dot-plots of FACS analyses and single cell sorting of fat samples. For every fat biopsy, the stromal vascular fraction was plated for 12 h in low serum conditions. Adherent cells were detached by quick trypsinization to obtain a cell preparation enriched for adipocyte progenitors. Dot plot of a representative DAPI staining to assess the numbers of living cells in the preparation is shown (top left). The treatment ensured a very high viability. An ISO-IgG control staining was performed to assess antibodies reactivity (top right). Pre-adipocytes isolated from SAT and VAT were stained with the hematopoietic-cell marker CD45 and the endothelial cell marker CD31 and selected from the double negative population as indicated in the gate P4. The double negative population was predominant in all biopsies (n= 9). However, the percentage of CD45 and CD31 positive cells was variable across samples, as can be appreciated in the SAT and VAT samples from the same donor that are shown in panel **f,** bottom left and right, respectively**.**

Fig. S2. Non-negative matrix factorization and comparison of extracted signatures to COSMIC cancer signatures and PCAWG single base signatures (SBS)

(**a**) Eight mutational signatures obtained from NMF of somatic mutation catalogues from healthy (n=161) and tumors (n=192) samples. For each combination of k (number of possible clusters, rows) and nFact (rank from NMF, columns) the general silhouette index (SI representing high reproducibility, values within the heatmap) was obtained. The chosen parameters were at nFact=8 and k=8 with SI=0.92. **b**) The 8 de novo signature profiles obtained from the NMF analysis were compared to already characterized signatures from COSMIC (30 signatures; http://cancer.sanger.ac.uk/cosmic/signatures) and PCAWG (60 signatures;[1]). Cosine measurements, indicating what COSMIC/PCAWG signature fits best with the de novo signatures, are provided. **c**) The mutational profiles of the 8 de novo signatures named after the most similar single base signature (SBS) from PCAWG

Fig. S3. Relative contribution of extracted signatures to healthy tissues and tissue-matched tumors

Relative contribution of the 8 mutational signatures to the somatic mutation catalogues of healthy (n=161) and tissue-matched tumor samples (n=192). Results of a statistical test (Mann-Whitney U test; *** FDR<1%,) testing enrichment of the exposure of the signature in one tissue compared to the same signature in all other tissues are shown in Table S3. Overall, our analysis shows that signatures SBS1, 3/8 and 5 were found ubiquitously and we defined this combination of signatures as “basal mutagenesis”. Consistent with this concept, cell types that were not common progenitors, had additional signatures that are associated with specific, mutagen exposure. Examples are 1) EP samples showing high levels of SBS7a, a signature induced by UV-light exposure, 2) the SkM cells used as a control for culture-induced mutagenesis in our previous study [2] (SkM-long), which showed SBS18, a signature linked to *in vitro*-culture stress [3, 4] and consequent production of intracellular reactive-oxygen species [5]. These samples were used as positive controls for prolonged exposure to a mutagen. All groups of cells were compared to these controls for either basal or mutagen-driven mutagenesis. SkinFB clustered in close proximity to the SkM-long samples (Figure 3a) and showed high levels of SBS18, consistent with the long *in vitro* culture required for the reprogramming protocol [6]. The SkinFB also showed the second highest SBS7a contribution after EP (Figure 3b). Intestine and colon stem cells formed a distinct cluster and were characterized by very high SBS1 contribution, previously explained with a high replication rate of these cells [7].

BLCA-ercc2del: bladder urothelial carcinoma with *ERCC2* knock out, BLCA: bladder urothelial carcinoma, EP: epidermis, Fb: skin fibroblasts, MELA-AU: melanoma, CLL-ES: chronic lymphocytic leukemia, SAT: subcutaneous fat, VAT: visceral fat, SkM: skeletal muscle, SkM_long: long-culture SkM cells, SARC: sarcoma, KT1: kidney tubule 1, KT2: kidney tubule 2, KICH: kidney chromophobe, KIRC: kidney renal clear cell carcinoma, KIRP: kidney renal papillary cell carcinoma, LHC: liver hepatocellular carcinoma, COAD: colon adenocarcinoma.

Fig. S4. Comparisons of cancer and normal samples

a

**a.** Comparison of somatic mutation profiles in tissue-matched healthy and cancer samples. The clustering (tSNE) based on the trinucleotide profile of somatic SNVs in the genome of healthy (n=161) and tumor (n=192) samples is shown. For each panel, different healthy and cancer samples are highlighted with specific colors (see legend), while all other samples are shown in grey. Cancer samples usually cluster in proximity of the tissue-matched healthy samples, but cancer and normal do not overlap. Bottom right panel shows the matching of two groups of healthy samples that shared a long culturing protocol: reprogrammed skin fibroblasts (SkinFB) and long-culture skeletal muscle progenitors (SkM-long). **b.-e.** Number of SNVs per genome, plotted according to age. Mutation burden (**b.)** and number of SBS5 mutations (**c.**) in normal kidney (KT2) and liver samples compared to cancer samples (bladder urothelial cell carcinoma) either NER proficient (BLCCs) or deficient (BLCC-*ERCC*del). The ERCCdel tumors were used as a control for SBS5 mutations induced by NER deficiency. Mutation burden (**d.**) and number of SBS40 mutations (**e.**) in normal kidney (KT1 and KT2) compared to kidney cancer samples of different subtypes: KICH (kidney chromophobe adenocarcinoma) KIRC (kidney clear cell renal cell carcinoma) KIRP (kidney renal papillary cell carcinoma).

Fig. S5. Comparison with mutation spectra determined by *in vitro* exposure to environmental agents

tSNE plot of the trinucleotide profile of somatic SNVs recovered in the genome of healthy cells (n=161), tumors (n=192) or an iPSC clone exposed to different environmental agents *in vitro* [3] (n=54). **a**. Environmental agents are highlighted with colors representing the different compound classes, while all other samples are shown in grey (normal: full dots, tumor: empty squares). A dashed line roughly describes the area occupied by common progenitors. The mutation spectrum of common progenitors does not show similarities with any spectra caused by environmental agent exposure, supporting the concept of basal mutagenesis. **b**. Same plot as in **a.**, but environmental agents are shown in grey, while normal (squares) and tumor (asterisks) genomes are shown in different colors according to the tissue of origin. The vast majority of spectra from treated cells located at the periphery of the plot and did not overlap with any normal or cancer genome (Figure S5a). Exceptions were 1) simulated solar radiation that perfectly overlapped with EP samples and one melanoma sample, 2) formaldehyde and alkylating agents, which located in proximity of KT2 and kidney tumors KIRC and KIRP (Figure S5b). Formaldehyde and multiple compounds with alkylating activity can be produced endogenously by human cells [8]. Therefore, the spectra of KT2, KIRP and KIRC might reflect the exposure of some kidney cells to endogenous formaldehyde and alkylating agents.

Fig. S6. Mutation enrichment in specific genomic regions in KT1, KT2, SAT, VAT samples and age-related differences

Enrichment (upward bars) or depletion (downward bars) of somatic mutations in indicated VEP genomic features or conserved regions in different tissue and age-groups. Kidney-1 (KT-1). kidney-2 (KT-2). subcutaneous fat (SAT) and visceral fat (VAT)

Fig. S7. Analysis of regional enrichment/depletion of mutations in different tissues

**a.-c.** Enrichment/depletion of mutations in specific genomic regions, as shown in figure 5a, but providing values either calculated separately for each tissue (**a.** common progenitors: SAT (N=22), VAT (N=20), SkM (N=29), blood (1 catalogue of mutations derived from randomly selected SNVs from multiple cell clones from the same individual)**-b**. intestinal stem cells: colon (N=21) intestine (N=14)) or from sample groups not shown in figure 5a (**c.** SkM-long (N=4), SkinFB (N=13)).The genomes were divided in multiple sectors (bins) according to decreasing DNA replication time (RT, bins 0 to 5, only bins 1, 3 and 5 are shown for clarity), increasing abundance of the histone mark H3K36me3 (bins 0-3), and increasing transcriptional levels (RNAseq, bins 0-3). The relative abundance of mutations in each bin vs bin 0 is estimated as the coefficient in negative binomial regression (expressed as log_2_), where error bars show its 95% C.I.

Common progenitors, including SAT, VAT, SkM and blood, but not KT1, showed the expected depletion of mutations with earlier RT, higher H3K36me3 abundance and higher transcription levels. This pattern indicates that the basal mutagenesis is actively counteracted by MMR and/or TC-NER. However, EP, KT2, KT1, liver, SkM-long and SkinFB deviated from the pattern seen for common progenitors and showed a loss of association of mutation rates with RT and H3K36me3. Therefore, in samples that appear to be exposed to a putative mutagen in addition to basal mutagenesis (Figure 3a and b), the early-replicating, active chromatin is less protected. These samples included the KT1 group, which showed a mutation profile similar to the common progenitors (Figure 4a), but also signs of cell damage (Figure 4f). Conversely, the intestinal stem cells (intestine and colon) showed regular association of mutations with RT and even stronger protection of H3K36me3-rich regions compared to common progenitors, suggesting that mutations in the active chromatin that are due to high proliferation are recognized by MMR.

Fig. S8. Association of mutations with replication timing in young and old genomes of healthy samples and MMR-proficient (MSS) or deficient (MSI) tumors

**a.** Enrichment/depletion of mutations according to DNA replication timing (RT) while controlling for CTCF ChipSeq peaks in either younger or older genomes as shown in figure 6c, but providing values calculated separately for each tissue. Enrichments are coefficients from negative binomial regression (as log2) and error bars are their 95% C.I **b.** Enrichment/depletions as in **a.** for 3 different groups of tumors (derived from colon, uterus, stomach) according to microsatellite stability. MSS= micro satellite stable, normal MMR function; MSI= micro satellite instability due to mutations in MMR genes which occurred with either early or late onset in the life of the patient. Fold-difference in depletion of mutations according to RT were 1.73 for MSS vs MSI-late and 2.13 for MSS vs MSI-early, showing that inactivation of MMR induces accumulation of mutations in early-replicating DNA that increases with time. These tumors were used as a control of the effect size of MMR-loss in causing mutations in early-replicating DNA. The fold-difference in young vs old healthy genomes (pulling together all tissues as in figure 6c) was 1.21, lower than that observed in MSI tumors, in agreement with only partial loss of MMR function with aging.

Supplementary Tables

Table S1. Donors and samples

**a.** Health status of six kidney donors included in the study

| donor ID | age | gender | general health | glomerular filtration rate (ml/min) | BMI (kg/m^2^) | History of  cancer | auto  immune diseases | tobacco consumption |
| --- | --- | --- | --- | --- | --- | --- | --- | --- |
| KD12 | 30 | M |  | 97 | 22 | no | no | non smoker |
| KD10 | 31 | M |  | 99 | 28 | no | no | non smoker |
| KD05 | 38 | F | asthma | 87 | 22 | no | no | 2-3 cigarettes/  day |
| KD09 | 63 | M | benign prostate hyperplasia history of cholecystectomy | 82 | 28 | no | no | not analyzed |
| KD11 | 66 | F | mild hyper tension history of hysterectomy | 74 | 20 | no | no | non smoker |
| KD06 | 69 | F | history of hysterectomy | 103 | 30 | no | no | 30 cigarettes/  day |

**b.** Clinical significance of germline variants detected in the kidney donors in 47 cancer genes*

| ClinVar  effect | Chr:Position | ID | Ref>Alt | Gene** | SweGen AF*** | gnomAD AF **** | KD12  AF | KD10  AF | KD05  AF | KD09  AF | KD11  AF | KD06  AF |
| --- | --- | --- | --- | --- | --- | --- | --- | --- | --- | --- | --- | --- |
| conflicting  uncertain | 1:45806053 | rs3219466 | G>A | MUTYH  5'prime UTR | 0.04 |  |  |  |  |  | 0.53 |  |
| uncertain | 2:47614013 | rs189199539 | G>C | EPCAM  3'prime UTR | na | 0 | 0.39 |  |  |  |  |  |
| conflicting uncertain | 2:48028273 | . | G>A | MSH6  missense | na | 0 |  |  | 0.55 |  |  |  |
| uncertain | 3:10193068 | rs141916278 | A>G | VHL  3'prime UTR | 0.006 |  |  | 0.5 |  |  |  |  |
| uncertain | 3:10194165 | . | CTTTTTT>C | VHL  downstream | na | 0.34 | 1 |  | 0.64 |  | 1 | 1 |
| conflicting  uncertain | 4:55524168 | rs140909964 | T>A | KIT  5'prime UTR | 0.006 |  |  | 0.63 |  |  |  |  |
| uncertain | 5:79950163 | rs70991108 | C>CTGGCGCGTCCCGCCCAGGT | DHFR  Sequence feature | 0.533 |  | 1 |  | 0.48 | 0.39 |  | 0.5 |
| uncertain | 8:90947409 | rs104895030 | G>C | NBN  3'prime UTR | 0.01 |  | 0.46 |  |  |  |  |  |
| conflicting uncertain | 10:88681437 | rs35619497 | C>T | BMPR1A  missense | 0.001 |  | 0.41 |  |  |  |  |  |
| uncertain | 10:88684422 | . | A>G | BMPR1A  3'prime UTR | na | na |  |  | 0.19 | 0.24 | 0.24 | 0.16 |
| uncertain | 11:108236786 | rs143531724 | T>C | ATM  3'prime UTR | 0.043 |  |  |  | 0.73 |  |  |  |
| uncertain | 12:58146061 | . | A>C | CDK4  5'prime UTR | na | 0.003 |  |  |  |  | 0.43 |  |
| conflicting  uncertain | 12:133253974 | rs61732929 | C>T | POLE  missense | 0.013 |  |  |  |  | 0.5 |  |  |
| uncertain | 14:95556508 | rs35649919 | CA>C | DICER1  3'prime UTR | na | 0.75 | 0.42 | 1 | 1 |  |  | 1 |
| risk factor | 16:68771034 | rs16260 | C>A | CDH1  upstream | 0.27 |  | 0.5 | 0.4 | 0.63 |  | 0.48 | 0.4 |
| uncertain | 16:68867611 | rs145920869 | C>CT | CDH1  3'prime UTR | na | 0.23 |  | 0.52 |  | 0.45 |  |  |
| conflicting | 17:7571752 | rs78378222 | T>G | TP53  3'prime UTR | 0.017 |  |  |  |  |  |  | 0.58 |
| uncertain | 17:7572154 | rs200757381 | GA>G | TP53  3'prime UTR |  | 0.61 | 1 | 0.33 | 0.68 | 0.53 | 0.68 |  |
| IMPACT=HiGH effect** | Chr:Position | ID | Ref>Alt | Gene | SweGen | gnomAD | KD12 | KD10 | KD05 | KD09 | KD11 | KD06 |
|  |  |  |  |  |  |  | AF | AF | AF | AF | AF | AF |
| splice donor variant | 2:47572363 | rs17036526 | G>C | EPCAM | 0.2060 | 0.1668 |  |  | 0/1 |  | 0/1 |  |
| frameshift variant & stop gained variant | 2:48033981 | rs267608142 | T>TTTGA | MSH6 | na | 0 | 0/1 |  |  |  |  |  |
| start lost | 2:215595645 | rs16852600 | C>T | BARD1 | 0.3505 | 0.2949 |  | 0/1 |  |  | 0/1 |  |
| splice acceptor variant | 10:89720633 |  | C>CT | PTEN | 0.1230 | 0.1708 | 0/1 |  |  |  | 0/1 |  |
| splice acceptor variant | 11:108121410 | rs34325032 | C>CT | ATM | 0.5185 | 0.3957 | 1/1 | 0/1 | 0/1 | 1/1 | 0/1 | 0/1 |
| splice acceptor variant | 13:32970097 | rs3072042 | C>CT | BRCA2 | 0.3450 | na |  |  | 1/1 |  |  |  |
| stop lost variant | 16:2087785 | rs34165865 | T>C | SLC9A3R2 | 0.1815 | 0.2040 |  | 0/1 |  | 0/1 |  | 0/1 |
| stop lost variant | 17:29705947 | rs2525574 | T>C | NF1 | 0.6080 | 0.5984 | 0/1 | 1/1 | 1/1 | 1/1 | 0/1 | 0/1 |

- Cancer genes included in the analysis are: *APC, ATM, AXIN2, BARD1, BMPR1A, BRCA1, BRCA2, BRIP1, CDH1, CDK4, CDKN2A, CHEK2, CTNNA1, DICER1, EPCAM, GREM1, HOXB13, KIT, MEN1, MLH1, MSH2, MSH3, MSH6, MUTYH, NBN, NF1, NTHL1, PALB2, PDGFRA, PMS2, POLD1, POLE, PTEN, RAD50, RAD51C, RAD51D, SDHA, SDHB, SDHC, SDHD, SMAD4, SMARCA4, STK11, TP53, TSC1, TSC2, VHL*

** annotation according to snpEff.

*** allele frequency of variants as reported in the Swedish population [9]

**** For variants not reported by SweGen the allele frequency in the Genome Aggregation Database, European (non-Finnish population) is reported (<https://www.biorxiv.org/content/10.1101/531210v2>).

**c.** Single cell cultures from kidney, epidermis, subcutaneous and visceral fat.

|  | | | | | |  |  |
| --- | --- | --- | --- | --- | --- | --- | --- |
|  | KT | EP | SAT | VAT | Individual | Gender | Age |
|  |  |  |  |  | KD12 | M | 30 |
| no. plated single cells | >8*10^5^ | >8*10^5^ | 288 | 192 |  |  |  |
| no. of colonies (14 days) | 36 | 0 | 35 | 11 |  |  |  |
| no. clones extracted DNA | 7 | 0 | 24 | 11 |  |  |  |
| no. sequenced clones | 5 | 0 | 5 | 5 |  |  |  |
|  |  |  |  |  |  |  |  |
|  |  |  |  |  | KD10 | M | 31 |
| no. plated single cells | >8*10^5^ | >8*10^5^ | 192 | 192 |  |  |  |
| no. of colonies (14 days) | 24 | 0 | 6 | 23 |  |  |  |
| no. clones extracted DNA | 7 | 0 | 6 | 10 |  |  |  |
| no. sequenced clones | 5 | 0 | 5 | 5 |  |  |  |
|  |  |  |  |  |  |  |  |
|  |  |  |  |  | KD05 | F | 38 |
| no. plated single cells | >8*10^5^ | >8*10^5^ | 192 | 0 |  |  |  |
| no. of colonies (14 days) | 9 | 0 | 38 | 0 |  |  |  |
| no. clones extracted DNA | 4 | 0 | 18 | 0 |  |  |  |
| no. sequenced clones | 3 | 0 | 5 | 0 |  |  |  |
|  |  |  |  |  |  |  |  |
|  |  |  |  |  | KD09 | M | 63 |
| no. plated single cells | >8*10^5^ | 0 | 0 | 0 |  |  |  |
| no. of colonies (7 days) | 11 | 0 | 0 | 0 |  |  |  |
| no. clones extracted DNA | 5 | 0 | 0 | 0 |  |  |  |
| no. sequenced clones | 5 | 0 | 0 | 0 |  |  |  |
|  |  |  |  |  |  |  |  |
|  |  |  |  |  | KD11 | F | 66 |
| no. plated single cells | >8*10^5^ | >8*10^5^ | 192 | 192 |  |  |  |
| no. of colonies (7 days) | 34 | 0 | 2 | 37 |  |  |  |
| no. clones extracted DNA | 8 | 0 | 2 | 15 |  |  |  |
| no. sequenced clones | 5 | 0 | 2 | 5 |  |  |  |
|  |  |  |  |  |  |  |  |
|  |  |  |  |  | KD06 | F | 69 |
| no. plated single cells | >8*10^5^ | >8*10^5^ | 192 | 192 |  |  |  |
| no. of colonies (7 days) | 24 | 2 | 13 | 18 |  |  |  |
| no. clones extracted DNA | 7 | 2 | 10 | 7 |  |  |  |
| no. sequenced clones | 5 | 2 | 5 | 5 |  |  |  |

**d.** Sequenced samples, coverage and somatic mutations

| Individual | Gender | Age | Clone name | Tissue | Coverage | SNVs | InDels | Normalized SNVs | Normalized InDels |
| --- | --- | --- | --- | --- | --- | --- | --- | --- | --- |
| KD12 | M | 30 | P4903_128 | KT | 0.92 | 359 | 45 | 390 | 49 |
|  |  |  | P4903_130 | KT | 0.93 | 317 | 23 | 342 | 25 |
|  |  |  | P4903_131 | KT | 0.93 | 402 | 43 | 433 | 46 |
|  |  |  | P4903_132 | KT | 0.92 | 443 | 36 | 480 | 39 |
|  |  |  | P4903_139 | SAT | 0.92 | 567 | 38 | 617 | 41 |
|  |  |  | P4903_140 | SAT | 0.92 | 630 | 50 | 685 | 54 |
|  |  |  | P4903_141 | SAT | 0.93 | 717 | 42 | 772 | 45 |
|  |  |  | P4903_142 | SAT | 0.83 | 364 | 42 | 438 | 51 |
|  |  |  | P4903_201 | SAT | 0.93 | 585 | 43 | 628 | 46 |
|  |  |  | P4903_133 | VAT | 0.93 | 665 | 41 | 718 | 44 |
|  |  |  | P4903_134 | VAT | 0.92 | 632 | 39 | 690 | 43 |
|  |  |  | P4903_135 | VAT | 0.93 | 709 | 56 | 764 | 60 |
|  |  |  | P4903_136 | VAT | 0.90 | 650 | 51 | 721 | 57 |
|  |  |  | P4903_137 | VAT | 0.87 | 602 | 42 | 693 | 48 |
|  |  |  |  |  |  |  |  |  |  |
| KD10 | M | 31 | P4903_101 | KT | 0.93 | 489 | 37 | 528 | 40 |
|  |  |  | P4903_102 | KT | 0.93 | 1019 | 115 | 1092 | 123 |
|  |  |  | P4903_103 | KT | 0.93 | 752 | 52 | 807 | 56 |
|  |  |  | P4903_104 | KT | 0.93 | 924 | 85 | 991 | 91 |
|  |  |  | P4903_105 | KT | 0.92 | 776 | 88 | 840 | 95 |
|  |  |  | P4903_106 | SAT | 0.93 | 640 | 53 | 688 | 57 |
|  |  |  | P4903_107 | SAT | 0.93 | 872 | 51 | 935 | 55 |
|  |  |  | P4903_108 | SAT | 0.93 | 771 | 51 | 833 | 55 |
|  |  |  | P4903_109 | SAT | 0.93 | 673 | 43 | 722 | 46 |
|  |  |  | P4903_110 | SAT | 0.93 | 574 | 41 | 618 | 44 |
|  |  |  | P4903_111 | VAT | 0.93 | 691 | 56 | 741 | 60 |
|  |  |  | P4903_112 | VAT | 0.92 | 647 | 41 | 705 | 45 |
|  |  |  | P4903_113 | VAT | 0.93 | 689 | 37 | 743 | 40 |
|  |  |  | P4903_114 | VAT | 0.93 | 635 | 40 | 684 | 43 |
|  |  |  | P4903_115 | VAT | 0.92 | 718 | 44 | 779 | 48 |
|  |  |  |  |  |  |  |  |  |  |
| KD05 | F | 38 | P4206_106 | KT | 0.93 | 865 | 82 | 934 | 89 |
|  |  |  | P4206_107 | KT | 0.93 | 1041 | 97 | 1118 | 104 |
|  |  |  | P4206_108 | KT | 0.92 | 967 | 100 | 1051 | 109 |
|  |  |  | P4206_101 | SAT | 0.93 | 755 | 34 | 810 | 36 |
|  |  |  | P4206_102 | SAT | 0.93 | 791 | 42 | 848 | 45 |
|  |  |  | P4206_103 | SAT | 0.93 | 708 | 40 | 759 | 43 |
|  |  |  | P4206_104 | SAT | 0.68 | 366 | 32 | 537 | 47 |
|  |  |  | P4206_105 | SAT | 0.83 | 610 | 57 | 735 | 69 |
|  |  |  |  |  |  |  |  |  |  |
| KD09 | M | 63 | P4206_126 | KT | 0.93 | 2265 | 209 | 2429 | 224 |
|  |  |  | P4206_127 | KT | 0.92 | 2103 | 195 | 2276 | 211 |
|  |  |  | P4206_128 | KT | 0.93 | 867 | 71 | 930 | 76 |
|  |  |  | P4206_129 | KT | 0.92 | 774 | 61 | 842 | 66 |
|  |  |  | P4206_130 | KT | Technical replicate of P4206_128 | | | | |
|  |  |  |  |  |  |  |  |  |  |
| KD11 | F | 66 | P4903_116 | KT | 0.92 | 850 | 106 | 928 | 116 |
|  |  |  | P4903_117 | KT | 0.93 | 2683 | 326 | 2888 | 351 |
|  |  |  | P4903_118 | KT | 0.92 | 2782 | 392 | 3036 | 428 |
|  |  |  | P4903_119 | KT | 0.93 | 1371 | 133 | 1482 | 144 |
|  |  |  | P4903_120 | KT | 0.91 | 2115 | 289 | 2325 | 318 |
|  |  |  | P4903_126 | SAT | 0.93 | 1286 | 101 | 1390 | 109 |
|  |  |  | P4903_127 | SAT | 0.91 | 1125 | 65 | 1241 | 72 |
|  |  |  | P4903_121 | VAT | 0.92 | 1302 | 82 | 1409 | 89 |
|  |  |  | P4903_122 | VAT | 0.93 | 1539 | 93 | 1655 | 100 |
|  |  |  | P4903_123 | VAT | 0.93 | 1418 | 81 | 1525 | 87 |
|  |  |  | P4903_124 | VAT | 0.90 | 1203 | 69 | 1331 | 76 |
|  |  |  | P4903_125 | VAT | 0.93 | 1418 | 86 | 1528 | 93 |
|  |  |  |  |  |  |  |  |  |  |
| KD06 | F | 69 | P4206_125 | EP | 0.93 | 1526 | 26 | 1639 | 28 |
|  |  |  | P4206_202 | EP | 0.93 | 1408 | 30 | 1510 | 32 |
|  |  |  | P4206_119 | KT | 0.93 | 1055 | 102 | 1131 | 109 |
|  |  |  | P4206_120 | KT | 0.93 | 2399 | 293 | 2570 | 314 |
|  |  |  | P4206_121 | KT | 0.93 | 3855 | 543 | 4132 | 582 |
|  |  |  | P4206_122 | KT | 0.93 | 3668 | 538 | 3933 | 577 |
|  |  |  | P4206_109 | SAT | 0.93 | 1160 | 59 | 1250 | 64 |
|  |  |  | P4206_110 | SAT | 0.93 | 1182 | 56 | 1274 | 60 |
|  |  |  | P4206_111 | SAT | 0.93 | 1445 | 91 | 1558 | 98 |
|  |  |  | P4206_113 | SAT | 0.92 | 1330 | 92 | 1438 | 99 |
|  |  |  | P4206_201 | SAT | 0.93 | 1108 | 71 | 1197 | 77 |
|  |  |  | P4206_114 | VAT | 0.93 | 1857 | 114 | 1995 | 122 |
|  |  |  | P4206_115 | VAT | 0.93 | 1983 | 118 | 2133 | 127 |
|  |  |  | P4206_116 | VAT | 0.93 | 1737 | 79 | 1867 | 85 |
|  |  |  | P4206_117 | VAT | 0.93 | 1727 | 109 | 1855 | 117 |
|  |  |  | P4206_118 | VAT | 0.93 | 1734 | 101 | 1860 | 108 |

**e.** Validation of somatic mutations with a technical replicate

|  | | |  |  |  |
| --- | --- | --- | --- | --- | --- |
| discovery set (clone) | validation set (clone) | n tested SNVs | % validated SNVs | n tested InDels | % validated InDels |
| P4206_128 | P4206_130 | 868 | **99.19** | 71 | **97.18** |
| P4206_128 | P4206_126 | 870 | 2.99 | 71 | 1.41 |
| P4206_128 | P4206_127 | 864 | 2.43 | 71 | 2.82 |
| P4206_128 | P4206_129 | 863 | 2.43 | 70 | 2.86 |
| P4206_128 | P4903_116 | 866 | 1.96 | 71 | 2.82 |
| P4206_128 | P4903_117 | 867 | 1.85 | 71 | 1.41 |
| P4206_128 | P4903_118 | 865 | 1.97 | 71 | 2.82 |
| P4206_128 | P4903_119 | 866 | 2.19 | 71 | 1.41 |
| P4206_128 | P4903_120 | 863 | 1.51 | 71 | 1.41 |

**a.** Tissue biopsies were obtained from living kidney donors (KD), a population of selected healthy individuals, subjected to extensive clinical analysis before surgery. None of the individuals presented a history of cancer or autoimmune disease. Other surgical interventions and diseases are reported in the “general health” column. **b.** Germline variants known to induce predisposition to cancer, as detected by the HaplotypeCaller in the blood of 6 kidney donors (KD12, KD10, KD05, KD09, KD11, KD06). Only 47 known, cancer-related genes were analyzed. Variants either reported in ClinVar or shwing a IMPACT=HIGH annotation from SnpEFF are shown. None of the patients showed variants annotated as “pathogenic” or “potentially pathogenic” by ClinVar.The table reports all variants with annotation “uncertain”, “conflicting_interpretations_of_pathogenicity” (conflicting) and “risk factor”. **c.** Cell culture data from kidney (KT=kidney tubular portion), epidermis (EP), subcutaneous adipose tissue (SAT) and visceral adipose tissue (VAT) biopsies obtained from six individuals included in the study. The number of plated single cells was registered for SAT and VAT tissues, which went through FACS sorting. FACS sorting of specific populations of kidney and epidermis cells was tested, but failed. Hence, colonies were obtained after direct plating of the unsorted fraction of cells isolated from KT and EP tissues. A minimum of 8*10^5^ viable cells were plated for each biopsy. **d.** Single cell genomes sequenced in the study were derived from 6 donors (first column) of different ages and 4 different tissues: kidney tubules (KT), epidermis (EP), subcutaneous adipose tissue (SAT) and visceral adipose tissue (VAT). Coverage indicates the percentage of autosomes covered by at least 15 reads in each sample. SNVs: single nucleotide variants. Indels: insertion deletions. SNVs and indels were normalized on coverage (last columns). **e.** To validate the somatic variants found in the study, the KT sample P4206_128 was grown in 2 different wells and sequencing data were obtained from both populations as a technical replicate. The percentage of somatic variants recovered in the discovery (P4206_128) and validation set (P4206_130) was registered. The same validation was tested in unrelated samples from the same (P4206_126/129) or a different biopsy (P4903_116/119) to test the unspecific background signal of the method when comparing two unrelated samples. Variants were called with our pipeline (see Supplementary Methods) in the discovery set and then tested in the validation set. Variants were considered validated when present in the validation set with a minimum coverage of 15x and a minimum of 3 reads supporting the alternative allele.

Table S2. Identity of samples used in the meta-analysis

| Sample type | # Clones | Median age (Range) | Individuals | Study |
| --- | --- | --- | --- | --- |
| BLCA  (bladder urothelial carcinoma) | 4 | 56 (43-84) | TCGA-FD-A3N6,TCGA-GD-A2C5,TCGA-BT-A20V,TCGA-DK-A1AE | TCGA WGS |
| BLCA-ercc2del | 4 | 72.5 (67-80) | TCGA-DK-A1A7,TCGA-FD-A3N5,TCGA-BT-A3PJ,TCGA-FT-A3EE | TCGA WGS |
| CLL-ES  (chronic lymphocytic leukemia,  Spanish cohort) | 5 | 58 (56-62) | DO52712,DO6438,DO52723,DO6370,DO52700 | ICGC WGS |
| COAD  (Colon adeno  carcinoma) | 26 | 68.5 (47-82) | TCGA-AA-A01S,TCGA-AD-A5EK,TCGA-A6-3807,TCGA-A6-A566,TCGA-QG-A5YW,TCGA-A6-A567,TCGA-A6-A56B,TCGA-AA-A01V,TCGA-QG-A5YX,TCGA-AA-A01T,TCGA-QG-A5YV,TCGA-AA-3956,TCGA-NH-A50T,TCGA-AA-3685,TCGA-AA-3994,TCGA-NH-A50V,TCGA-QG-A5Z1,TCGA-A6-2680,TCGA-A6-2681,TCGA-AA-A02Y,TCGA-AA-3664,TCGA-AA-3529,TCGA-AA-3534,TCGA-AA-A01X,TCGA-AA-3514,TCGA-AA-A02O, | TCGA WGS |
| KICH  (kidney chromophobe adenocarcinoma) | 34 | 46.5 (17-86) | TCGA-KO-8406,TCGA-KN-8422,TCGA-KO-8405,TCGA-KO-8417,TCGA-KN-8431,TCGA-KM-8440,TCGA-KN-8429,TCGA-KL-8341,TCGA-KN-8418,TCGA-KO-8416,TCGA-KN-8424,TCGA-KN-8437,TCGA-KO-8407,TCGA-KN-8419,TCGA-KL-8342,TCGA-KM-8438,TCGA-KL-8327,TCGA-KM-8476,TCGA-KN-8421,TCGA-KN-8426,TCGA-KN-8425,TCGA-KN-8427,TCGA-KL-8325,TCGA-KM-8477,TCGA-KL-8323,TCGA-KO-8411,TCGA-KN-8435,TCGA-KL-8346,TCGA-KN-8434,TCGA-KM-8639,TCGA-KL-8326,TCGA-KO-8409,TCGA-KO-8410,TCGA-KN-8432 | TCGA WGS |
| KIRC  (Kidney  Clear cell renal cell carcinoma) | 38 | 61.5 (40-84) | TCGA-BP-4968,TCGA-BP-4807,TCGA-CJ-4899,TCGA-CJ-5681,TCGA-B0-5693,TCGA-A3-3370,TCGA-A3-3387,TCGA-CJ-4639,TCGA-A3-3363,TCGA-A3-3324,TCGA-B2-4101,TCGA-BP-4326,TCGA-CJ-6033,TCGA-BP-4977,TCGA-CJ-4870,TCGA-CJ-5682,TCGA-CZ-5987,TCGA-B0-5695,TCGA-B2-4102,TCGA-AK-3428,TCGA-B0-5094,TCGA-BP-4756,TCGA-CZ-4856,TCGA-BP-5010,TCGA-CZ-5454,TCGA-A3-3372,TCGA-CJ-4885,TCGA-CJ-4918,TCGA-DV-5566,TCGA-AK-3455,TCGA-CJ-4878,TCGA-CW-6093,TCGA-BP-4327,TCGA-BP-5168,TCGA-A3-3308,TCGA-BP-4781,TCGA-B2-4099,TCGA-AK-3454 | TCGA WGS |
| KIRP  (Kidney renal papillary cell carcinoma) | 16 | 58.5 (41-79) | TCGA-AL-3466,TCGA-B3-3925,TCGA-AL-3467,TCGA-B1-A47O,TCGA-AL-3473,TCGA-B3-4103,TCGA-AL-3472,TCGA-HE-A5NF,TCGA-A4-A57E,TCGA-B3-3926,TCGA-GL-A4EM,TCGA-B1-A47N,TCGA-B9-4113,TCGA-B9-A44B,TCGA-B3-4104,TCGA-B1-A47M | TCGA WGS |
| LIHC  (Liver hepatocellular carcinoma) | 10 | 52 (23-76) | TCGA-DD-A1EH,TCGA-CC-5261,TCGA-DD-A1EI,TCGA-EP-A2KB,TCGA-CC-A1HT,TCGA-ES-A2HT,TCGA-BC-A216,TCGA-DD-A1E9,TCGA-BC-A10Q,TCGA-DD-A1EG | TCGA WGS |
| MELA-AU  (Melanoma, Australian cohort) | 15 | 60 (41-90) | DO220848,DO222377,DO220863,DO222859,DO222820,DO222832,DO222464,DO222802,DO222810,DO222738,DO222368,DO222591,DO222453 | ICGC WGS |
| SARC  (Sarcoma) | 40 | 60.5 (41-82) | TCGA-N1-A6IA,TCGA-DX-A3LW,TCGA-DX-A3U8,TCGA-IW-A3M4,TCGA-X2-A95T,TCGA-DX-A240,TCGA-DX-A3U5,TCGA-FX-A3NJ,TCGA-IE-A4EK,TCGA-DX-A23R,TCGA-FX-A48G,TCGA-DX-A48U,TCGA-DX-A1L0,TCGA-HB-A43Z,TCGA-HB-A5W3,TCGA-WP-A9GB,TCGA-DX-A1L3,TCGA-DX-A2IZ,TCGA-DX-A2J4,TCGA-DX-A3LU,TCGA-DX-A2J0,TCGA-FX-A2QS,TCGA-DX-A3LT,TCGA-DX-A3U6,TCGA-IS-A3K7,TCGA-K1-A3PN,TCGA-FX-A3RE,TCGA-DX-A3LY,TCGA-DX-A1KW,TCGA-DX-A3U7,TCGA-IE-A4EI,TCGA-IS-A3KA,TCGA-MO-A47R,TCGA-IW-A3M5,TCGA-DX-A1L2,TCGA-DX-A3LS,TCGA-DX-A3M1,TCGA-IF-A4AJ,TCGA-DX-A1KU,TCGA-DX-A6Z2 | TCGA WGS |
| Blood  progenitors | 1 | 59 (59-59) |  | Lee-Six [10] |
| Skin  Fibroblasts  (reprogrammed) | 13 | 7 (6-15) | i03-04,i07-03,i03-03,iS1120-03,iS1123-03 | Abyzov [6] |
| Colon  stem cells | 21 | 56 (9-67) | donor1,donor2,donor3,donor4,donor18,donor19 | Blokzijl [7] |
| Intestine  stem cells | 14 | 44.5 (3-87) | donor5,donor6,donor7,donor8,donor9,donor10, donor11, donor12, donor13 | Blokzijl [7] |
| Liver  stem cells | 10 | 46 (30-55) | donor14,donor15,donor16,donor17,donor18 | Blokzijl [7] |
| SkM  (skeletal muscle progenitors) | 29 | 63 (21-78) | CES1,CES2,CES3,CES4,CES6,CES7,CES8 | Franco [2] |
| SkM_long  (long-culture SkM progenitors) | 4 | 26 (26-26) | CES9 | Franco [2] |

Table S3. Differential contribution of specific signatures in different tissue-groups

| Tissue | SBS1 | SBS2 | SBS3 | SBS5 | SBS7a | SBS17b | SBS18 | SBS40 |
| --- | --- | --- | --- | --- | --- | --- | --- | --- |
| Blood | 0.3766 | 0.3339 | 0.9374 | 0.3281 | 0.2885 | 0.4351 | 0.1643 | 0.1950 |
| KT1 | 0.9653 | 0.0385 | 0.1182 | **0.0076** | 0.6106 | 0.4611 | 0.0846 | 0.8631 |
| SAT | 0.2147 | **0.0010** | **1.72E-05** | 0.3620 | 0.0265 | 0.4505 | 0.0166 | 0.0430 |
| SkM | **0.0056** | **0.0005** | **7.57E-07** | 0.0846 | **3.72E-05** | 0.0191 | 0.2020 | **0.0085** |
| VAT | 0.1182 | 0.0862 | **1.16E-05** | 0.4268 | 0.8631 | 0.0832 | **0.0045** | 0.2329 |
| KT2 | **0.0010** | 0.0521 | 0.4429 | **3.70E-05** | 0.4191 | 0.0696 | **0.0010** | **5.40E-04** |
| Liver | 0.0563 | 0.9604 | 0.3064 | **2.15E-05** | 0.0224 | 0.6723 | 0.7329 | 0.8325 |
| Colon | **1.08E-11** | **0.0010** | **2.23E-08** | 0.9653 | 0.7314 | **0.0002** | 0.1508 | **0.0025** |
| Intestine | **6.79E-08** | **0.0086** | **1.28E-04** | 0.3766 | 0.9374 | 0.1500 | 0.4427 | **0.0045** |
| EP | 0.1526 | 0.0410 | 0.0383 | 0.0438 | 0.0379 | 0.0379 | 0.0519 | 0.0379 |
| SkM  long | 0.0505 | 0.0831 | 0.1182 | 0.0126 | 0.0383 | 0.2971 | **0.0048** | 0.5001 |
| Fb | **7.57E-07** | 0.9653 | 0.6570 | 0.3648 | 0.0788 | 0.4303 | **0.0001** | 0.8661 |
| MELA  AU | 0.3339 | 0.2280 | 0.4117 | 0.7880 | **0.0011** | 0.3384 | 0.0843 | 0.5626 |
| SARC | **6.15E-04** | **2.63E-15** | **2.08E-10** | **0.0010** | 0.2848 | 0.8139 | 0.9018 | 0.9018 |
| KICH | **1.72E-05** | **0.0059** | 0.7921 | 0.0113 | 0.7043 | **0.0008** | **0.0093** | 0.4611 |
| KIRC | **3.46E-07** | 0.2484 | 0.9514 | **0.0040** | **0.0019** | 0.0262 | 0.0505 | **2.63E-15** |
| KIRP | **1.03E-03** | 0.4755 | 0.0278 | 0.3110 | 0.7001 | **0.0032** | 0.4107 | **0.0003** |
| LIHC | **0.0010** | 0.3577 | 0.7036 | **0.0002** | 0.9007 | 0.3900 | **0.0009** | 0.3585 |
| BLCA | 0.2436 | **0.0027** | 0.2769 | 0.0110 | 0.6301 | 0.3766 | 0.9495 | 0.3404 |
| BLCA ercc2del | 0.1015 | **0.0085** | **0.0088** | 0.1176 | 0.2329 | 0.4665 | 0.1358 | 0.9653 |
| CLLE_ES | 0.9514 | 0.0110 | 0.1077 | 0.1222 | 0.2969 | 0.0160 | 0.6660 | 0.4500 |
| COAD | **6.50E-07** | 0.5700 | **4.54E-09** | **6.79E-08** | **7.57E-07** | **5.38E-06** | **1.06E-13** | 0.0440 |

FDR q-values of data shown in Figure S3 (exposure of different healthy and cancer tissue-groups to extracted SBS). To test significantly higher or lower contribution of each signature in the different tissue-groups, Mann-Whitney U test was performed. For each signature, the relative contribution obtained for all clones in one tissue was compared to the relative contribution in all other tissues. For example, all KT1 clones were compared to all other clones but KT1. Then, false discovery rate (FDR) was used to adjust the p value for multiple testing. FDR values (q values)< 0.01 are shown in bold. Red colored FDR values indicate higher contribution of the specified signature in the tissue.

Table S4. Age-related SBS signature changes in healthy samples

1. **Yearly SNV increase for SBS signatures and tissue group**

|  | Common Progenitors | | KT2 | | Liver | | Intestinal | |
| --- | --- | --- | --- | --- | --- | --- | --- | --- |
|  | Estimate+  Std.Error | adj P-value | Estimate+ Std.Error | adj P-value | Estimate+ Std.Error | adj P-value | Estimate+ Std.Error | adj P-value |
| SBS1 | 2.41±0.4 | 2,7E-04 | 1.12±0.38 | 0,050 | 2.18±1.02 | 0,250 | 13.92±1.55 | 1,4E-06 |
| SBS2 | 0.18±0.09 | 2,6E-01 | 2.36±0.58 | 0,027 | 0.45±1.42 | 1,000 | 0.83±0.14 | 8,6E-05 |
| SBS3/8 | 3.5±0.77 | 2,2E-03 | 8.52±2.56 | 0,063 | 6.27±2.51 | 0,144 | 1.5±0.44 | 2,8E-02 |
| SBS5 | 3.72±0.48 | 5,5E-06 | 10.54±2.6 | 0,039 | 10.31±3.47 | 0,048 | 3.6±0.53 | 4,3E-05 |
| SBS7a | 0.24±0.2 | 1,0E+00 | 1.5±1.5 | 1,000 | 2.53±0.65 | 0,023 | 1.55±0.28 | 7,5E-04 |
| SBS17 | 0.65±0.1 | 2,5E-04 | 2.64±0.99 | 0,138 | 1.38±0.4 | 0,074 | 0.4±0.09 | 8,5E-03 |
| SBS18 | 0.91±0.18 | 1,1E-03 | 2.08±0.99 | 0,280 | 0.75±0.88 | 1,000 | 2.99±0.85 | 1,2E-02 |
| SBS40 | 0.26±0.2 | 8,1E-01 | 13.52±4.37 | 0,088 | 4.21±2.32 | 0,366 | 1.09±0.35 | 3,3E-02 |

**b. Fraction of mutations attributed to specific SBS signatures for different tissue and age-groups**

|  | Common Progenitors | | KT2 | | Liver | | Intestinal | |
| --- | --- | --- | --- | --- | --- | --- | --- | --- |
|  | young | old | young | old | young | old | young | old |
| SBS1 | 0.18± 0.01 | 0.19±0.01 | 0.07±0.01 | 0.04±0.01 | 0.07±0.01 | 0.08±0.02 | 0.57±0.03 | 0.56±0.01 |
| SBS2 | 0.02±0 | 0.02±0 | 0.02±0.01 | 0.04±0 | 0.06±0.02 | 0.04±0.01 | 0.02±0.01 | 0.03±0 |
| SBS3/8 | 0.37± 0.01 | 0.34±0.01 | 0.22±0.03 | 0.2± 0.02 | 0.17±0.04 | 0.18±0.02 | 0.05±0.02 | 0.06±0.01 |
| SBS5 | 0.2± 0.01 | 0.25±0.01 | 0.31±0.03 | 0.3± 0.03 | 0.37±0.02 | 0.35±0.04 | 0.11±0.02 | 0.12±0.01 |
| SBS7a | 0.07± 0.01 | 0.04±0 | 0.11±0.02 | 0.06±0.01 | 0.08±0.01 | 0.08±0 | 0.07±0.01 | 0.06±0 |
| SBS17b | 0.02± 0 | 0.04±0 | 0.04±0.01 | 0.06±0.01 | 0.03±0.01 | 0.03±0.01 | 0.02±0 | 0.02±0 |
| SBS18 | 0.07± 0.01 | 0.07±0.01 | 0.04±0.01 | 0.04±0.01 | 0.08±0.01 | 0.07±0.01 | 0.12±0.02 | 0.12±0.01 |
| SBS40 | 0.07± 0.01 | 0.04±0.01 | 0.2± 0.02 | 0.26±0.02 | 0.15±0.04 | 0.17±0.02 | 0.05±0.01 | 0.04±0.01 |

**a.** Estimated SNV increase and error calculated using linear mixed model and P-values adjusted for multiple testing using Bonferroni correction. Common progenitors (KT1, SAT, VAT and SkM clones, n=82), KT2 (n=14), liver (n=10), intestinal (colon and intestine clones, n=35). **b.** Fraction of mutations attributed to each signature. Mean values and SEM are shown for each signature in younger (<50 years) and older (>50 years) samples of different tissue groups. Common progenitors (KT1, SAT, VAT and SkM clones, young n=45, old n=37), KT2 (young n=5, old n=9 ), liver (young n=7, old n=4), intestinal (colon and intestine clones, young n=14, old n=21).

Table S5. Genes commonly mutated in KIRC/KIRP and SNVs, InDels and CNVs detected in 69 progenitor cells from healthy KT, SAT, VAT, EP

**a. InDels and SNVs**

| gene | chr | position | ref | alt | location | individual | clone | group | CADD score |
| --- | --- | --- | --- | --- | --- | --- | --- | --- | --- |
| MTOR | 1 | 11225812 | A | T | gene | KD06 | P4206_122 | KT2 | 11,1 |
| MTOR | 1 | 11249816 | C | T | exon | KD11 | P4903_127 | KT1,SAT,VAT | 15,9 |
| MTOR | 1 | 11260051 | T | C | gene | KD05 | P4206_101 | KT1,SAT,VAT | 1,0 |
| BAP1 | 3 | no SNVs/InDels detected | | | | | | | |
| PBRM1 | 3 | 52604466 | A | G | gene | KD06 | P4206_121 | KT2 | 7,0 |
| PBRM1 | 3 | 52680481 | TTA | T | gene | KD11 | P4903_126 | KT1,SAT,VAT | 3,3 |
| PBRM1 | 3 | 52608492 | G | A | gene | KD06 | P4206_117 | KT1,SAT,VAT | 1,4 |
| SETD2 | 3 | 47194034 | C | A | gene | KD12 | P4903_131 | KT1,SAT,VAT | 0,4 |
| SETD2 | 3 | 47060608 | T | C | gene | KD11 | P4903_117 | KT2 | 0,7 |
| SETD2 | 3 | 47207097 | G | GT | gene | KD11 | P4903_117 | KT2 | 1,3 |
| VHL | 3 | no SNVs/InDels detected | | | | | | | |
| MET | 7 | 116324863 | C | T | gene | KD12 | P4903_133 | KT1,SAT,VAT | 0,2 |
| MET | 7 | 116335938 | T | C | gene | KD11 | P4903_122 | KT1,SAT,VAT | 8,3 |
| MET | 7 | 116378331 | A | G | gene | KD12 | P4903_139 | KT1,SAT,VAT | 7,0 |
| MET | 7 | 116432781 | A | T | gene | KD05 | P4206_106 | KT1,SAT,VAT | 1,0 |
| MET | 7 | 116432985 | A | T | gene | KD06 | P4206_113 | KT1,SAT,VAT | 1,7 |
| CDKN2A | 9 | no SNVs/InDels detected | | | | | | | |
| PTEN | 10 | 89692729 | G | A | gene | KD06 | P4206_202 | EP | 1,7 |
| PTEN | 10 | 89692733 | T | G | gene | KD06 | P4206_116 | KT1,SAT,VAT | 0,8 |
| TP53 | 17 | no SNVs/InDels detected | | | | | | | |

**b. CNVs**

| gene | chr | gene region | cnv region | copy number | individual | clone | group |
| --- | --- | --- | --- | --- | --- | --- | --- |
| MTOR | 1 | 11166592-11322564 | 14930-17291861 | 1 | KD05 | P4206_102 | KT1,SAT,VAT |
| MTOR | 1 | 11166592-11322564 | 10671323-11220809 | 1 | KD05 | P4206_105 | KT1,SAT,VAT |
| BAP1 | 3 | 52435029-52444366 | 52279594-52569098 | 1 | KD05 | P4206_104 | KT1,SAT,VAT |
| SETD2 | 3 | 47057919-47205457 | 47110722-47113754 | 1 | KD05 | P4206_104 | KT1,SAT,VAT |
| VHL | 3 | 10182692-10193904 | 10188798-10199901 | 1 | KD05 | P4206_104 | KT1,SAT,VAT |
| BAP1 | 3 | 52435029-52444366 | 52030607-52569098 | 1 | KD05 | P4206_105 | KT1,SAT,VAT |
| PBRM1 | 3 | 52579368-52719933 | 52704032-52758570 | 1 | KD05 | P4206_105 | KT1,SAT,VAT |
| SETD2 | 3 | 47057919-47205457 | 46862874-47110228 | 1 | KD05 | P4206_105 | KT1,SAT,VAT |
| SETD2 | 3 | 47057919-47205457 | 47110722-47113754 | LOH | KD05 | P4206_105 | KT1,SAT,VAT |
| VHL | 3 | 10182692-10193904 | 9766580-10527762 | 1 | KD05 | P4206_105 | KT1,SAT,VAT |
| BAP1 | 3 | 52435029-52444366 | 60596-75616017 | 3 | KD11 | P4903_118 | K2 |
| PBRM1 | 3 | 52579368-52719933 | 60596-75616017 | 3 | KD11 | P4903_118 | K2 |
| SETD2 | 3 | 47057919-47205457 | 60596-75616017 | 3 | KD11 | P4903_118 | K2 |
| VHL | 3 | 10182692-10193904 | 60596-75616017 | 3 | KD11 | P4903_118 | K2 |
| BAP1 | 3 | 52435029-52444366 | 60596-75608687 | 3 | KD11 | P4903_119 | K2 |
| PBRM1 | 3 | 52579368-52719933 | 60596-75608687 | 3 | KD11 | P4903_119 | K2 |
| SETD2 | 3 | 47057919-47205457 | 60596-75608687 | 3 | KD11 | P4903_119 | K2 |
| VHL | 3 | 10182692-10193904 | 60596-75608687 | 3 | KD11 | P4903_119 | K2 |
| VHL | 3 | 10182692-10193904 | 9975247-10233821 | 3 | KD12 | P4903_142 | KT1,SAT,VAT |
| MET | 7 | no CNVs detected | | | | | |
| CDKN2A | 9 | no CNVs detected | | | | | |
| PTEN | 10 | no CNVs detected | | | | | |
| TP53 | 17 | no CNVs detected | | | | | |

Transformation of kidney cells and consequent tumor initiation occurs as a consequence of specific genetic events, including deletion of the chromosome 3p and mutations in the tumor suppressor genes VHL, PBRM1, BAP1, SETD2, PTEN or the oncogene MTOR for KIRC (clear cell renal cell carcinoma), and gain of chromosomes 7 and 17 and mutations of the oncogene MET for KIRP (papillary renal cell carcinoma). A screening of the loci containing these genes was performed in 69 progenitor cells from healthy KT, SAT, VAT and EP, in order to compare the SNVs, InDels (**a**.) and CNVs (**b**.) present in the KT2 and the KT1-SAT-VAT groups. Pathogenicity of InDels and SNVs was predicted with CADD (Phred scores are shown in the last column. Scores <15 correspond to minor effects on the protein sequence). CNVs were detected with ASCAT (see also Supplementary figure 7 and Supplementary Table 9).

Table S6. Chromosomal aberrations detected in 69 progenitor cells from healthy KT, SAT, VAT, EP and 29 skeletal muscle progenitors

| Individual | Age (years) | Clone | Tissue | Chromosomal aberration |
| --- | --- | --- | --- | --- |
| KD11 | 66 | P4903_118 | KT-2 | gain 3 |
|  |  | '' | '' | gain X |
|  |  | P4903_119 | '' | gain 3 |
|  |  | P4903_120 | '' | gain 12 |
|  |  |  |  |  |
| KD06 | 69 | P4206_121 | KT-2 | gain 4q21-q27 |
|  |  | '' | '' | loss X |
|  |  | P4206_119 | KT-1 | gain 13 |
|  |  | P4206_117 | VAT | loss of 8q24 |
|  |  |  |  |  |
|  |  |  |  |  |
| Individual | Age (years) | Clone | Tissue | Chromosomal aberration |
| CES6 | 78 | P2703_126 | SkM | gain 10 |
|  |  | P2703_130 | SkM | gain 21 |
|  |  |  |  |  |
| CES2 | 75 | P4206_203 | SkM | loss X |

Macroscopic chromosomal gains and losses detected with ASCAT.

**Supplementary bibliography**

1. Alexandrov L.B. JK, Nicholas J Haradhvala, Mi Ni Huang, Alvin W T Ng, Arnoud Boot, Kyle R Covington, Dmitry A Gordenin, Erik Bergstrom, Nuria Lopez-Bigas, Leszek, J Klimczak, JohnR McPherson, Sandro Morganella, Radhakrishnan Sabarinathan, David A Wheeler, Ville Mustonen, Gad Getz, Steven G. Rozen, Michael R Stratton, PCAWG Mutational Signatures Working Group, ICGC/TCGA Pan-Cancer Analysis of Whole Genomes Net: **The Repertoire of Mutational Signatures in Human Cancer.** *bioRxive* 2018.

2. Franco I, Johansson A, Olsson K, Vrtacnik P, Lundin P, Helgadottir HT, Larsson M, Revechon G, Bosia C, Pagnani A, et al: **Somatic mutagenesis in satellite cells associates with human skeletal muscle aging.** *Nat Commun* 2018, **9:**800.

3. Kucab JE, Zou X, Morganella S, Joel M, Nanda AS, Nagy E, Gomez C, Degasperi A, Harris R, Jackson SP, et al: **A Compendium of Mutational Signatures of Environmental Agents.** *Cell* 2019.

4. Zou X, Owusu M, Harris R, Jackson SP, Loizou JI, Nik-Zainal S: **Validating the concept of mutational signatures with isogenic cell models.** *Nat Commun* 2018, **9:**1744.

5. Rouhani FJ, Nik-Zainal S, Wuster A, Li Y, Conte N, Koike-Yusa H, Kumasaka N, Vallier L, Yusa K, Bradley A: **Mutational History of a Human Cell Lineage from Somatic to Induced Pluripotent Stem Cells.** *PLoS Genet* 2016, **12:**e1005932.

6. Abyzov A, Tomasini L, Zhou B, Vasmatzis N, Coppola G, Amenduni M, Pattni R, Wilson M, Gerstein M, Weissman S, et al: **One thousand somatic SNVs per skin fibroblast cell set baseline of mosaic mutational load with patterns that suggest proliferative origin.** *Genome Res* 2017, **27:**512-523.

7. Blokzijl F, de Ligt J, Jager M, Sasselli V, Roerink S, Sasaki N, Huch M, Boymans S, Kuijk E, Prins P, et al: **Tissue-specific mutation accumulation in human adult stem cells during life.** *Nature* 2016, **538:**260-264.

8. Nakamura J, Mutlu E, Sharma V, Collins L, Bodnar W, Yu R, Lai Y, Moeller B, Lu K, Swenberg J: **The endogenous exposome.** *DNA Repair (Amst)* 2014, **19:**3-13.

9. Ameur A, Dahlberg J, Olason P, Vezzi F, Karlsson R, Martin M, Viklund J, Kahari AK, Lundin P, Che H, et al: **SweGen: a whole-genome data resource of genetic variability in a cross-section of the Swedish population.** *Eur J Hum Genet* 2017, **25:**1253-1260.

10. Lee-Six H, Obro NF, Shepherd MS, Grossmann S, Dawson K, Belmonte M, Osborne RJ, Huntly BJP, Martincorena I, Anderson E, et al: **Population dynamics of normal human blood inferred from somatic mutations.** *Nature* 2018, **561:**473-478.
