# Supplementary material for: Whole genome DNA sequencing provides an atlas of somatic mutagenesis in healthy human cells and identifies a tumor-prone cell type
Source: Genome Biol. 2019 Dec 18;20:285. doi: 10.1186/s13059-019-1892-z (PMC6918713; doi:10.1186/s13059-019-1892-z)
Supplement: Supplementary file 4 — Additional file 4. Review history [file 13059_2019_1892_MOESM4_ESM.docx]

Review History

**First round of review**

**Reviewer 1**

**Are you able to assess all statistics in the manuscript, including the appropriateness of statistical tests used?**

Yes, and I have assessed the statistics in my report.

**Comments to author:**

In this manuscript, 69 single genomes from four healthy tissue types from six donors were analyzed using in vitro clonal expansion of single cells and whole-genome sequencing (WGS). Based on this data, somatic mutagenesis patterns were compared across tissue types and donor ages. The analysis suggested that somatic mutagenesis is composed of basal and mutagen-driven processes, and especially, that kidney cells can be divided into two types KT1 and KT2. Among those KT2 is differentiated towards the proximal tubule and predisposes to cancer driver mutations, potentially caused by exposure of different mutagen compared with KT1. MMR and NER repair processes appear to be declined in the genome of healthy cells upon ageing. This is a well-written manuscript with some intriguing research findings. I have several comments that the reviewers should address when revising this manuscript.

Major comment 1: Figure 1 (Page 6, line 1)

Regarding the Figure 1 and experimental design, the reason why the authors chose four tissue type (Human kidney-tubules (KT), epidermis (EP), subcutaneous and visceral adipose tissue (SAT and VAT)) among the variety of tissue types in the human body is not well defined. In terms of embryonic lineage, KT has endodermal origin, adipose tissue (SAT and VAT) is derived from mesodermal in origin, and EP is originated from Ectoderm, which means four tissue type covers all three embryonic lineages. However, each of three lineages (endoderm, mesoderm, and ectoderm) give rise to a broad spectrum of tissues including blood, muscle, brain, or lung. The selection criteria of tissue/cell types included in the study should be better defined in the manuscript.

Major comment 2: Figure1 (Page 6, line 21)

In this study, two to five single genomes per biopsy were sequenced from six individuals of either younger or older age. Even though those single genomes were sequenced with high coverage (15X), it is hard to represent heterogeneous human tissue mosaicism with two to five single cells per biopsy. The authors should clarify and discuss what fraction of the somatic mutational spectra are expected to be represented by selected single genomes per biopsy compared with bulk WGS from the same biopsy.

Major comment 3: Page 7, line 7, "The tissue of origin of a cell is not the main determinant of the somatic mutation profile"

In Figure 2a, the provided heat map nicely shows that there is a cluster shared by multiple tissues (KT1, SAT, VAT), but kidney cells were divided into two distinct clusters which support the conclusion of this paragraph (The tissue of origin is not the main determinant of the somatic mutation profile). However when the authors extended analysis to include public datasets from skin fibroblasts, stem cells from liver, intestine, colon, skeletal muscle, and blood, Figure 3a shows that some of the clusters in the t-SNE plot are separated by their tissue type (Colon and intestine, Skin fibroblast) even though one cluster is shared by multiple tissue types (common progenitors). It can be interpreted as tissue of origin is not the only factor but it can contribute to somatic mutation accumulation. Therefore in the title of the paragraph in Page 7, it would be great to highlight two distinct spectra in the kidney tissue rather than to generalize the observation to a broad spectrum of tissues.

In addition to this, to be clear I certainly do not regard the conclusion that cell type is a more direct determinant for mutagenesis patterns than tissue type as unexpected. As the authors state in the introduction already, several cell type-specific genomic features, such as histone modification and DNA replication findings, have been shown to affect patterns of somatic mutation. Why is it a surprise that cell type is a better determinant for mutation profiles than tissue type? The authors should revise their manuscript to not overstate the difference between their findings and "prior presumptions".

Major comment 4: Figure 4 (Page 10, line 57), "Overall, our somatic mutation data and phenotypic characterization show that, rather than two strictly separated cell types, KT1 and KT2 appear as a continuum of phenotypes ranging from uncommitted kidney tubule progenitors (KT1) to more committed PT epithelial cells (KT2)."

In this paragraph, the authors investigated the potential cell-types of KT1 and KT2 using qPCR of known molecular markers of kidney cell types. KT1 represents a progenitor-like and KT2 a more differentiated proximal tubule (PT) cell type. Their analysis nicely shows distinct molecular characteristic of KT1 and KT2 cells, but the identity, localization, and potential microenvironment and mutagens of KT1 were not very clear compared to KT2. The analysis showed the expression of progenitor markers in KT1, previous studies showed that progenitor cells with CD133 and CD24 positive have the potential to give rise to multiple cell types in the kidney (PMID: 19092120). Current single-cell transcriptome of kidney provided comprehensive cell type profiles and their differentiation trajectories (PMID: 30449713). Especially, it provides single-cell transcriptome from early tubule progenitors, loop of Henle (LOH) cells, to mature proximal tubule cells. It would be a great addition to the study if the authors could profile the transcriptomes of KT1 and KT2 clones and project them to those differentiation trajectories defined by single-cell transcriptome to further characterize KT1 identity.

Major comment 5: Figure 5e (Page 12, line15)

In Figure 5e, the authors showed that KT2 exhibits an intriguing enrichment of mutations in regulatory regions while KT1, SAT, VAT showed depletion of mutation in those regions. Could the authors compare KT1, SAT, VAT, KT2 separately, to demonstrate differences between KT1 and KT2 which originate from the same tissue?

Minor comment 1: In the introduction, authors have mentioned that "It is possible to obtain WGS data relative to a single genome while avoiding single cell-sequencing. This method requires in vitro clonal expansion of a single cell prior to sequencing to select somatic variants that were present in vivo and eliminate those that occurred during culture." How are somatic variants occurring during culture being eliminated? Please describe in the methods or result section.

Minor comment 3: Figure 6a (Page 12, line 45)

In Figure 6a, Enrichment/depletion of mutations in specific genomic regions was compared based on the multiple sectors defined by DNA replication time, H3K36me3, and RNA-seq expression level. However, it was unclear whether cell-type matched external data were downloaded, or whether the authors performed experiments on the cell-type matched samples. If publicly available data was used, the source of the data needs to be mentioned in the manuscript. Also, KT1 and KT2 clones might have different epigenome and transcriptome profile. It needs to be clarified whether RNA-seq data from bulk kidney or KT1 and KT2 specific RNA-seq data were compared for this figure.

Minor comment 4: Figure 7c (Page 14, line 18)

In Figure 7c, enrichment of mutations according to the replication timing bins and Ctcf peaks was depicted and different age groups were compared. Older genomes showed a weakened association of mutations with RT compared to younger ones, indicating a partial loss of MMR activity according to the manuscript. However, the figure also shows strengthened association of mutations with Ctcf cohesion peaks compared to younger ones. The description and importance of this phenomena were not mentioned in the manuscript. Further description and interpretation in the manuscript would be needed for this ctcf associations.

**Reviewer 2**

**Are you able to assess all statistics in the manuscript, including the appropriateness of statistical tests used?**

No, I do not feel adequately qualified to assess the statistics.

**Comments to author:**

The article "Whole genome DNA sequencing provides an atlas of somatic mutagenesis in healthy

human cells and identifies a tumor-prone cell type" from Franco et al. aim to elucidate the somatic mutations differences in different non-cancer cell types in order to provide a somatic mutation atlas for human cells and potentially gain insight into pathogenesis of cancer.

The study findings are interesting but difficult to follow in their presentation, and even though a lot of data were generated, it would benefit from cutting down the narrative significantky and focusing on the most salient findings. The methodology is very sparsely described.

1. Human kidney-tubules, epidermis, subcutaneous and visceral adipose tissue from healthy

individuals of different ages were selected for the general aim of "explore differences in mutagenic processes occurring in adult human tissues". The study winds up focusing on a kidney tubular cells. Was there any novel insight gained rom adipose tissue, besides their role as comparison points?

2. The methods about the utilized strategy for single-cell sequencing and somatic variants identification are very sparsely described and require more details to inform the reader. The strategy used by authors is basically to expand in-vitro a clonal cell from the original biopsied tissue through 17-20 passages. The resulting population underwent WGS analysis and the results interpreted as single-clone derived. This approach was elaborated in order to avoid single-cell WGS, but is potentially confounded by mutations that may take place during the in-vitro expansion phase. The methods and QC results do not adequately address this issue, which is of concern given that cells were sequenced after 17-20 passages. The references cited all acknowledge this issye as a limiations of the approach, but this is not clearly stated in this paper. [Abyzov, A. et al.. Genome Res. 201, or Behjati, S. et al. Nature 2014). The authors excluded chromosomes X and Y from the analyses but in fact these may serve as good controls as it may be possible to examine the variant allele frequencies on the X and Y chromosome in males to get an idea of the cell-culture associated mutation rate. Deviations from 100% would suggest culture induced artifact.

2- Somatic variant calling section states "First, the variants present in any of the SCCs were gathered in a comprehensive list of interesting positions, which was specific to each individual.", how exactly were these "interesting positions" identified and which one were excluded? Based on what criteria?

3. Clonal expansion. Why only 2 lines from the same individual could be analyzed? It is not clear and may represent a strong bias for this population somatic mutation results. Moreover, why FACS sorting failed in KT and not in AT?

4. Patients. Patients are defined as "healthy" and "with no history of cancer". Nonetheless, 2 patients reported an history of hysterectomy, why it was operated? This may be important as it represents a surgical operations generally related to cancer/hyperplasia. Also the prostate benign hyperplasia in patient KD09 should be stated in text.

5. From the clustering results as depicted in figure 2A and 3A, it is difficult to tell the degree of confidence leading to the separation into the two main clusters. This is important because the authors claim this proves the "tissue specific independence" of the somatic mutations "in contrast to the current dogma" (pag. 17, line 36). A such strong assumption needs careful evaluation and analysis across much larger datasets of normal tissues. I would also provide better labeling of sample colors for Figure 3A to highlight position of KT2 cells, as it can be confused with colon profiles.

6. The samples were selected from young and old individuals, so the dichotomous distribution is imperfect for derivation of a linear curve to calculate for accumulation of mutations. The lines in figure 3D are drawn post hoc so I would be more cautious in the interpretation of such data.

7. The authors test should acknowledge limitations due to multiple hypothesis testing, when they looking at 8 mutational signatures across each cell type. Some form of permutation procedure should be used to demonstrate the robustness of assigning a specific mutation signature to a specific cell type.

8. Based on the scant characterization of these cells, I would be hesitant to call KT1 "uncommitted progenitor cells" and "committed epithelial cells".

The identity of KT1 and KT2 clones should be confirmed with a wider panel of markers. While AQP1 and PDZK1 are a good start, they are expressed in other segments of the nephron, so it would be important to check additional markers such as LRP2 or SLC9A3 or thers from recent single cell maps of the kidney.

9- While the individuals contributing cells were healthy, they may still harbor pathogenic mutations that would influence mutation process. Since 2-5% of the population may harbor a germline predisposition to cancer, it would be important to perform an annotation of germline variants that could be linked to cancer.

**Authors Response**

**Point-by-point responses to the reviewers’ comments:**

Point-by-point answer to reviewers

Reviewer #1: In this manuscript, 69 single genomes from four healthy tissue types from six donors were analyzed using in vitro clonal expansion of single cells and whole-genome sequencing (WGS). Based on this data, somatic mutagenesis patterns were compared across tissue types and donor ages. The analysis suggested that somatic mutagenesis is composed of basal and mutagen-driven processes, and especially, that kidney cells can be divided into two types KT1 and KT2. Among those KT2 is differentiated towards the proximal tubule and predisposes to cancer driver mutations, potentially caused by exposure of different mutagen compared with KT1. MMR and NER repair processes appear to be declined in the genome of healthy cells upon ageing. This is a well-written manuscript with some intriguing research findings. I have several comments that the reviewers should address when revising this manuscript.

Major comment 1: Figure 1 (Page 6, line 1)

Regarding the Figure 1 and experimental design, the reason why the authors chose four tissue type (Human kidney-tubules (KT), epidermis (EP), subcutaneous and visceral adipose tissue (SAT and VAT)) among the variety of tissue types in the human body is not well defined. In terms of embryonic lineage, KT has endodermal origin, adipose tissue (SAT and VAT) is derived from mesodermal in origin, and EP is originated from Ectoderm, which means four tissue type covers all three embryonic lineages. However, each of three lineages (endoderm, mesoderm, and ectoderm) give rise to a broad spectrum of tissues including blood, muscle, brain, or lung. The selection criteria of tissue/cell types included in the study should be better defined in the manuscript.

*AR: This is an interesting comment but we did not base our choice of tissues on developmental features. Conversely, we have a specific interest in the mechanisms of aging and our current and past research has mainly focused on skin, fat, kidney and skeletal muscle, all tissues where the tissue degeneration coupled with aging leads to clear morphological changes. This work aims to define the relationship between loss of genome integrity and morphological tissue changes during aging. The scientific reasons for choosing these four tissues among all others have been stated in the results section, page 6, first paragraph.*

Major comment 2: Figure1 (Page 6, line 21)

In this study, two to five single genomes per biopsy were sequenced from six individuals of either younger or older age. Even though those single genomes were sequenced with high coverage (15X), it is hard to represent heterogeneous human tissue mosaicism with two to five single cells per biopsy. The authors should clarify and discuss what fraction of the somatic mutational spectra are expected to be represented by selected single genomes per biopsy compared with bulk WGS from the same biopsy.

*AR: The limitations of sequencing a small number of genomes per tissue have been acknowledged in the introduction, page 3, last paragraph. We agree with the reviewer that the analysis of a very small cell N per tissue prevents the discovery of specific mutations that might be important for the tissue-homeostasis of that specific biopsy. However, our analyses focus on the number of mutations/genome and mutation patterns, rather than specific mutations. When looking at these types of data (mutation burden and patterns), the variability among different cells of the same biopsy is very low, and the same happens for cells from different biopsies of the same tissue. This low variability indicate that we are capturing properties shared by the majority of the cells, even though we analyze a very small N of samples. This property of the data has been acknowledged on page 4, second paragraph.*

Major comment 3: Page 7, line 7, "The tissue of origin of a cell is not the main determinant of the somatic mutation profile"

In Figure 2a, the provided heat map nicely shows that there is a cluster shared by multiple tissues (KT1, SAT, VAT), but kidney cells were divided into two distinct clusters which support the conclusion of this paragraph (The tissue of origin is not the main determinant of the somatic mutation profile). However when the authors extended analysis to include public datasets from skin fibroblasts, stem cells from liver, intestine, colon, skeletal muscle, and blood, Figure 3a shows that some of the clusters in the t-SNE plot are separated by their tissue type (Colon and intestine, Skin fibroblast) even though one cluster is shared by multiple tissue types (common progenitors). It can be interpreted as tissue of origin is not the only factor but it can contribute to somatic mutation accumulation. Therefore in the title of the paragraph in Page 7, it would be great to highlight two distinct spectra in the kidney tissue rather than to generalize the observation to a broad spectrum of tissues.

*AR: This is a valid concern. We agree with the referee that the tissue of origin is an important contributor to the mutation spectrum, as highlighted in fig 3a, and that the title of the paragraph illustrating fig 2a can underestimate its importance. For these reasons, the title of the paragraph has been modified and now reads: “The tissue of origin of a cell is not the only determinant of the somatic mutation profile”. The conclusion of the paragraph has been modified and now reads: “we identify a stereotyped mutation spectrum in multiple, different tissues (KT, SAT, VAT) and two distinct spectra in the same tissue (KT1 and KT2), suggesting that the tissue of origin is not the main determinant of somatic mutation accumulation in this sample set”*

In addition to this, to be clear I certainly do not regard the conclusion that cell type is a more direct determinant for mutagenesis patterns than tissue type as unexpected. As the authors state in the introduction already, several cell type-specific genomic features, such as histone modification and DNA replication findings, have been shown to affect patterns of somatic mutation. Why is it a surprise that cell type is a better determinant for mutation profiles than tissue type? The authors should revise their manuscript to not overstate the difference between their findings and "prior presumptions".

*AR: This is a misunderstanding and we have tried to rephrase this paragraph to avoid to overstate our findings. To our knowledge, our manuscript is the first one that provides evidence of two different mutation profiles in the same tissue portion and attributes it to rather subtle cellular characteristics. We believe that our work increases the resolution of the link between tissue-specific mutagens and somatic mutagenesis. To stress this concept and avoid the overstatement of cell-type vs tissue-type as a better determinant for somatic mutations we modified the sentence of the introduction directly stating this. The sentence now reads: “The findings derived from our atlas of somatic mutations in healthy tissues do not support a simple association of each tissue to a specific somatic mutation pattern. In contrast, we identify a stereotypical, mutational pattern across progenitor cells from a variety of tissues and two distinct mutation profiles in the same tissue portion, indicating that mutagen exposure is modulated by multiple factors besides tissue type. In particular, we identify cell differentiation state and cell-type-specific activities as critical determinants of somatic mutagenesis.”*

Major comment 4: Figure 4 (Page 10, line 57), "Overall, our somatic mutation data and phenotypic characterization show that, rather than two strictly separated cell types, KT1 and KT2 appear as a continuum of phenotypes ranging from uncommitted kidney tubule progenitors (KT1) to more committed PT epithelial cells (KT2)."

In this paragraph, the authors investigated the potential cell-types of KT1 and KT2 using qPCR of known molecular markers of kidney cell types. KT1 represents a progenitor-like and KT2 a more differentiated proximal tubule (PT) cell type. Their analysis nicely shows distinct molecular characteristic of KT1 and KT2 cells, but the identity, localization, and potential microenvironment and mutagens of KT1 were not very clear compared to KT2. The analysis showed the expression of progenitor markers in KT1, previous studies showed that progenitor cells with CD133 and CD24 positive have the potential to give rise to multiple cell types in the kidney (PMID: 19092120). Current single-cell transcriptome of kidney provided comprehensive cell type profiles and their differentiation trajectories (PMID: 30449713). Especially, it provides single-cell transcriptome from early tubule progenitors, loop of Henle (LOH) cells, to mature proximal tubule cells. It would be a great addition to

the study if the authors could profile the transcriptomes of KT1 and KT2 clones and project them to those differentiation trajectories defined by single-cell transcriptome to further characterize KT1 identity.

*AR: We thank the referee for suggesting a very interesting approach to better characterize the KT1 population. However, multiple technical issues prevented us from trying this experiment. First, the suggested paper (PMID: 30449713) acknowledges the difficulties to compare the transcriptome of cells derived from the in vitro differentiation of organoids with adult kidney cells (like our clones), stating that organoid-derived cells are more similar to immature/embryonic kidney cells. Second, and more critical, RNA extracted from our kidney clones is derived from cells that have been cultured in 2D and without specific factors for 3-5 weeks and we are convinced that this treatment has changed the general transcription profile. We are afraid that a global analysis, like RNA seq, will be biased more by the culture conditions than by the cell type, making the interpretation of these data and comparison to data from external sources very difficult.*

*Since the availability of RNA from every clone is very scarce, we rather preferred a targeted analysis of selected genes that can be meaningful for defining KT1 and KT2. In the revised version of the manuscript we have thereby expanded our list of gene expression profiles by qPCR including 8 additional genes. This analysis is now presented in figure 5d and led to subtle, but important changes in our classification of KT1 and KT2. For an extended discussion of the new data please see the answer to Reviewer 2, point 8.*

Major comment 5: Figure 5e (Page 12, line15)

In Figure 5e, the authors showed that KT2 exhibits an intriguing enrichment of mutations in regulatory regions while KT1, SAT, VAT showed depletion of mutation in those regions. Could the authors compare KT1, SAT, VAT, KT2 separately, to demonstrate differences between KT1 and KT2 which originate from the same tissue?

*AR: The requested figure was included in the supplement to the previous submission. Each group (KT1, KT2, SAT, VAT) was shown separately in Figure S6. This figure also shows the comparison between genomes from young and old donors.*

Minor comment 1: In the introduction, authors have mentioned that "It is possible to obtain WGS data relative to a single genome while avoiding single cell-sequencing. This method requires in vitro clonal expansion of a single cell prior to sequencing to select somatic variants that were present in vivo and eliminate those that occurred during culture." How are somatic variants occurring during culture being eliminated? Please describe in the methods or result section.

*AR: We apologize for the omission of this important description. This topic was discussed in our previous publication (Franco et al., Nat Communications, 2018) and therefore we referred to it and did not explained the somatic mutation calling strategy in details here. Reviewers´ comments made us realize that this is an important issue for the paper. In accordance also with referee´s 2 requests, the methods have been moved to main text and extended and the filtering strategy, as well as our validation strategy to exclude the presence of culture-induced variants, have been mentioned in the first paragraph of results.*

*The main reasons why we are confident that our catalogues of somatic mutations do not contain variants occurred during the cell culture are the following:*

*1) the filtering strategy allows a very strict range of allele frequency (0.4-0.6, this is now stated in the results section, page 6, line 16) stricter than in other publications that used the same strategy (Abyzov A., et al, Genome Res, 2017; Blockzijl F. et al., Nature, 2016). Additional filterings are: a minimum coverage of the position of 15x, and a minimum of 3 reads supporting the alternative alleles. All together, these filtering criteria make extremely unlikely for a variant occurred during culture to be included in our list. In fact, variants that occurred during culture would be present only in a subpopulation of the cells. Unless mutations were non-neutral, a mutation must have occurred during the first cell division to yield an allele frequency of 0.25 and in the second cell division to yield AF 0.12. We only allow mutations with AF 0.4-0.6. We acknowledge that it is possible, even though unlikely, that a portion of the variants occurred during the first divisions is inaccurately sequenced with allele frequency 0.4-0.6. But since variants occurring in a genome in every cell division have been estimated to range between 2 and 10 (Lynch M. , PNAS, 2010, 107: 961 ; Zong, C., et al. Science, 2012; 338:1622) and our lists of somatic variants contain 300 to 3900 SNVs, the impact of such variants on our lists can be considered negligible.*

*2) to validate our strategy experimentally, we analyzed the mutation pattern in a set of “positive control” samples for culture-induced variants, e.g., skeletal-muscle cell clones obtained from cells that stayed 50 days in culture before the cloning and were subsequently cultured as regular SkM cell clones. These long-culture SkM cells showed features known to be associated with cell culture-induced mutations, such as a high fraction of C>A conversions and the cancer signature 18 (Franco et al, Nat Communications, 2018 and this manuscript). These features were absent in the other cell types that followed the standard culturing procedure, thus confirming that our strategy for calling somatic variants efficiently excludes mutations that occurred during in vitro culture and provides a faithful representation of the genome of the SC at the moment of the isolation from the tissue.*

*3) We present in this manuscript a validation of SNVs and InDels that has been carried on a kidney clone that was cultured in 2 different wells (Table S1E, samples P4206_128 and P4206_130). 99 and 97% of somatic SNVs and InDels called in sample P4206_128 were found also in P4206_130, demonstrating that culture-induced mutations cannot constitute more than 1-3% of variants in our lists. Similar results were obtained following identical validation strategy (98.8% of SNVs and 98.5 % of InDels where validated in the same clone cultured in a different well) in our published paper (Franco et al, Nat Communications, 2018).*

Minor comment 3: Figure 6a (Page 12, line 45)

In Figure 6a, Enrichment/depletion of mutations in specific genomic regions was compared based on the multiple sectors defined by DNA replication time, H3K36me3, and RNA-seq expression level. However, it was unclear whether cell-type matched external data were downloaded, or whether the authors performed experiments on the cell-type matched samples. If publicly available data was used, the source of the data needs to be mentioned in the manuscript. Also, KT1 and KT2 clones might have different epigenome and transcriptome profile. It needs to be clarified whether RNA-seq data from bulk kidney or KT1 and KT2 specific RNA-seq data were compared for this figure.

*AR: We apologize for the omission. The datasets of chromatin marks and gene expression used for the panel 6a have been downloaded from the Roadmap Epigenomics project. The source of the data is now explicit in the methods. It is hard to find deposited data that are a good or even reasonable match for the different cell types analyzed. Moreover, data largely overlap in different tissue types. For these reasons, and to simplify the analyses, the same epigenomic and transcriptomic data were applied to all cell types. These were created as an average of all cell types available, as explained in the methods.*

Minor comment 4: Figure 7c (Page 14, line 18)

In Figure 7c, enrichment of mutations according to the replication timing bins and Ctcf peaks was depicted and different age groups were compared. Older genomes showed a weakened association of mutations with RT compared to younger ones, indicating a partial loss of MMR activity according to the manuscript. However, the figure also shows strengthened association of mutations with Ctcf cohesion peaks compared to younger ones. The description and importance of this phenomena were not mentioned in the manuscript. Further description and interpretation in the manuscript would be needed for this ctcf associations.

*Binding sites for CTCF and Cohesin show a peculiar pattern of association with mutations. This has mainly been characterized in certain tumor types and the mechanisms still need to be elucidated. However, a publication by Katainen et al., Nat Genet 2015, found that the accumulation of mutations in CTCF-Cohesin peaks is found only in tumors that have a functional MMR. Overall, the fact that old genomes show low number of mutations in these regions is an additional evidence in support of MMR-defects with increased age. This discusson has been added in the last section of results, page 14, line 8*

Reviewer #2: ===

The article "Whole genome DNA sequencing provides an atlas of somatic mutagenesis in healthy human cells and identifies a tumor-prone cell type" from Franco et al. aim to elucidate the somatic mutations differences in different non-cancer cell types in order to provide a somatic mutation atlas for human cells and potentially gain insight into pathogenesis of cancer.

The study findings are interesting but difficult to follow in their presentation, and even though a lot of data were generated, it would benefit from cutting down the narrative significantly and focusing on the most salient findings.

*AR: We thank the reviewer for the overall positive comment on our findings. The paragraphs: “An atlas of somatic mutagenesis in healthy tissues distinguishes basal and mutagen-driven processes”, and “Different efficiency of DNA repair in cells exposed to basal mutagenesis or additional mutagens” have been shortened in order to keep the focus exclusively on original results. Interesting observations from the meta-analysis have been moved to the legend of the corresponding supplementary figures.*

The methodology is very sparsely described.

*The method section, previously divided between main text and supplementary, has been unified and extended and can be now found in main text.*

1. Human kidney-tubules, epidermis, subcutaneous and visceral adipose tissue from healthy individuals of different ages were selected for the general aim of "explore differences in mutagenic processes occurring in adult human tissues". The study winds up focusing on a kidney tubular cells. Was there any novel insight gained rom adipose tissue, besides their role as comparison points?

*AR: Visceral and subcutaneous fat are considered two different tissues and undergo very different morphological changes during aging. Visceral fat generally increases, while subcutaneous fat is depleted. Our aim was to explore a possible contribution of somatic mutagenesis to this phenotype. This hypothesis has now been stated in the first section of results (page 6, first paragraph). However, we found that mutation profiles in SAT and VAT are indistinguishable. Therefore our data do not support a major role of somatic mutagenesis in the differences mentioned above. This is now stated in the discussion (page 18, line 27).*

2. The methods about the utilized strategy for single-cell sequencing and somatic variants identification are very sparsely described and require more details to inform the reader. The strategy used by authors is basically to expand in-vitro a clonal cell from the original biopsied tissue through 17-20 passages. The resulting population underwent WGS analysis and the results interpreted as single-clone derived. This approach was elaborated in order to avoid single-cell WGS, but is potentially confounded by mutations that may take place during the in-vitro expansion phase. The methods and QC results do not adequately address this issue, which is of concern given that cells were sequenced after 17-20 passages. The references cited all acknowledge this issue as a limitation of the approach, but this is not clearly stated in this paper. [Abyzov, A. et al.. Genome Res. 201, or Behjati, S. et al. Nature 2014). The authors excluded chromosomes X and Y from the analyses but in fact these may serve as good controls as it may be possible to examine the variant allele frequencies on the X and Y chromosome in males to get an idea of the cell-culture associated mutation rate. Deviations from 100% would suggest culture induced artifact.

*AR: Our method for detection of high confidence somatic variants is principally based on a strict filtering on the allele frequency, stricter than in other publications that used the same method (Abyzov A., et al, Genome Res, 2017; Blockzijl F. et al., Nature, 2016). Variants showing the AF required by our filtering criteria (0.4-0.6) are greatly enriched for variants that are heterozygous in the clone and this condition is necessarily achieved only by variants that are shared by all cells of the clone. Variants that occurred from the first division of the clone onwards are supposed to show AF<0.25 and be excluded from our lists (see also answer to Reviewer 1, major comment 5). Somatic variants called in autosomes were validated in multiple ways in this and our previous publication (Franco et al, Nat Communication 2018) demonstrating that the fraction of culture-induced mutations in our lists is not higher than 1-3% (see answer to Reviewer 1, major comment 5). Chromosome X and Y were excluded from the analysis, because it is impossible to apply the same filtering on AF. To call variants with a different AF filtering we would have needed a specific validation. The methods concerning this part have been clarified and extended. Specific reference to our stringent filtering on AF has been added in the first section of results and the method paragraph clearly highlights our validation strategy as a mean to confirm the absence of culture-induced variants in our lists.*

2- Somatic variant calling section states "First, the variants present in any of the SCCs were gathered in a comprehensive list of interesting positions, which was specific to each individual.", how exactly were these "interesting positions" identified and which one were excluded? Based on what criteria?

*AR: We apologize for the poor description of the method and hope that the revised version is clearer. Here is a brief clarification of the specific questions from the reviewer: to avoid missing variants, we initially called all variants present in each clone with 3 different callers (HaplotypeCaller (GATK), MuTect2 (GATK 3.5.0) and FermiKit version r178), this is the unfiltered list of “interesting positions”. From this huge list we wanted to extract only high confidence somatic variants, which are defined as heterozygous in the clone and almost absent in an unrelated tissue (blood bulk, in this case). We had multiple clones from the same individual that needed to be filtered using the same blood bulk. For this reason we compiled a list of interesting positions that is the union of all the lists obtained from each clone of the same individual and processed the clones together. We finally reported the variants for each clone that passed the filtering and were NOT found in other clones, because variants in common between clones of the same individual were often found to be germline variants not called in the blood bulk.*

*In summary, “the list of interesting position” does NOT exclude any variant that was called by either HaplotypeCaller, MuTect2 or FermiKit. The filtering is downstream of this list and is mainly based on AF. The “list of interesting positions” was used as a track to search for the AF of each variant in the relative .bam files. The criteria to filter and retain only high confidence somatic variants are listed in the method section.*

3. Clonal expansion. Why only 2 lines from the same individual could be analyzed? It is not clear and may represent a strong bias for this population somatic mutation results.

*AR: We think that the above comment probably refers to our EP samples. To answer this comment: we tried to culture keratinocytes from 5 individuals, but the protocol was rather inefficient in our hands. We managed to obtain only 2 single cell clones from individual KD06 (the oldest in the cohort). It is possible that those clones managed to proliferate in culture because they had transformed properties. However, karyotype was normal and we could not find specific genetic lesions linked to cancer (Table S5 and S6). We decided to include the two EP clones for selected analyses, such as the somatic mutation profile and distribution of mutations, because they showed exactly the patterns that are expected from epidermal cells exposed to UV-light. For example, figure S5 shows perfect overlap between these 2 clones and cells exposed to simulated sunlight in vitro.*

Moreover, why FACS sorting failed in KT and not in AT?

*This is a technical problem depending on the kidney biopsies: we obtained our biopsies from healthy individual donating one of their kidneys to a diseased person. In these cases, the transplanted kidney can be analyzed for clinical and scientific purposes, but, to ensure not to damage the precious organ, only a very small biopsy can be taken. The tiny biopsy (7-8 mg of tissue) from the kidney cortex contains a very restricted number of cells. In addition, the CD24/CD133 double positive cells are supposed to be <4% of renal cells (Angelotti et al, Stem Cells, 2012). The single cell FACS procedure is very precise, but leads to the loss of the majority of positive cells contained in the sample. Therefore, to be successful, this procedure requires a cell population much bigger than the one we could obtain from the biopsies. For example, the same procedure results efficient for obtaining CD24/CD133 double positive cells from bigger biopsies (e.g. normal tissue surrounding cancer explants or post mortem tissue, as in Bussolati B. Am J Pathol, 2005 and Bussolati B., Am J Physiol Renal Physiol, 2012). Conversely, the fat tissue that we obtained from surgery was material that the surgeon would have removed and trashed anyway. We obtained huge biopsies in some cases and consequent high number of progenitors to stain and sort. It is possible that also the protocol for culturing EP progenitors was limited by the size of the biopsy.*

4. Patients. Patients are defined as "healthy" and "with no history of cancer". Nonetheless, 2 patients reported an history of hysterectomy, why it was operated? This may be important as it represents a surgical operations generally related to cancer/hyperplasia. Also the prostate benign hyperplasia in patient KD09 should be stated in text.

*One hysterectomy was not due to any form of hyper-proliferative condition (uterine prolapse), while the other was due to myomas, a benign tumor, extremely common in the female population during fertile age. The prostate benign hyperplasia is a condition found in 50% of men aged >60. The presence of these benign forms of hyperplasia has now been stated in the first section of results and better specified in Table S1.*

5. From the clustering results as depicted in figure 2A and 3A, it is difficult to tell the degree of confidence leading to the separation into the two main clusters. This is important because the authors claim this proves the "tissue specific independence" of the somatic mutations "in contrast to the current dogma" (pag. 17, line 36). A such strong assumption needs careful evaluation and analysis across much larger datasets of normal tissues. I would also provide better labeling of sample colors for Figure 3A to highlight position of KT2 cells, as it can be confused with colon profiles.

*The statement about "tissue specific independence" of the somatic mutations "in contrast to the current dogma" has been changed according to Reviewer´s 1 major comment 3 (see page 17, line 24) and the title and conclusions of the second paragraphs of results have been mitigated, also in agreement with Reviewer´s 1 comments. However, we calculated the distance of each tissue from the other ones showed in the hierarchical clustering of figure 2a.*

*Sample1 EP KT1 KT2 SAT VAT*

*EP 0,003*

*KT1 0,701 0,237*

*KT2 0,606 0,332 0,177*

*SAT 0,651 0,240 0,368 0,170*

*VAT 0,704 0,225 0,371 0,166 0,138*

*This numeric representation of distances shows that KT2 is similarly distant from KT1, SAT and VAT (numbers highlighted in red), while the distances internal to the KT1, SAT and VAT group are smaller (highlighted in yellow), in agreement with our conclusions.*

6. The samples were selected from young and old individuals, so the dichotomous distribution is imperfect for derivation of a linear curve to calculate for accumulation of mutations. The lines in figure 3D are drawn post hoc so I would be more cautious in the interpretation of such data.

*AR: We acknowledge this limitation in the discussion (page 16, line 18). However, a linear increase of mutation burden with age has been so far demonstrated as a good approximation for all tissues, using both tumor and normal-cell data (Milholland B. et al, Exp Gerontol, 2017). It is true that we are the first group to report the somatic mutations in healthy kidney and fat and its relation to aging, but our analyses suggest that the age-increase of mutations is in line with that registered in other studies from healthy cells so far. This is a graph showing all data from an extended meta-analysis. Data from our individuals are highlighted in colors representing each tissue, while data from other studies are shown in grey.*

*Meta-analysis from:*

*Colon, intestine and liver: Blockzijl F., Nature, 2016*

*HSPCs (hematopoietic progenitor cells): Osorio FG, Cell Reports, 2018*

*B-lymphocytes: Zhang L., PNAS, 2019*

*Skin: Sainin N., PLoS genetics. 2016*

*Colorectal: Lee-Six H., bioRxiv. 2018:416800.*

*brain PFC (prefrontal cortex) and hippocampus: Lodato MA, Science, 2018*

*esophagus: Martincorena I., Science, 2018*

*endometrium: Moore L., bioRxiv. 2018:505685.*

7. The authors test should acknowledge limitations due to multiple hypothesis testing, when they looking at 8 mutational signatures across each cell type. Some form of permutation procedure should be used to demonstrate the robustness of assigning a specific mutation signature to a specific cell type.

*AR: Eight mutational signatures were obtained through a robust permutation test, now better described in the methods: “Briefly, the SNVs from the healthy samples and the tumor samples were analyzed jointly, where a NMF (non-negative matrix factorization) analysis was applied to matrices of mutation counts across the 96 mutational contexts, as customary (see e.g. [16]). Upon repeated runs (n=200) of the NMF procedure (function nmf in the R package NMF, using the default “Brunet” algorithm) on the bootstrap-resampled mutation count data, the 200 NMF results were clustered using k-medoids algorithm (function pam in R package cluster) to obtain the final set of mutational signatures and their contributions (exposures) in every sample. The signature profiles obtained from this NMF analysis were compared using cosine similarity to the known mutational signatures”. However, we agree with the reviewer that some of our conclusions about enrichment of specific mutational signatures in specific cell types compared to the others included in the analyses would be better supported by a statistical test that acknowledges multiple hypothesis testing. To this end, we tested the contribution of each signature in each tissue presented in figure S3. A table reporting all the FDR-adjusted-p values from Mann-Whitney U test (q values) has been added (Table S3).*

8. Based on the scant characterization of these cells, I would be hesitant to call KT1 "uncommitted progenitor cells" and "committed epithelial cells".

The identity of KT1 and KT2 clones should be confirmed with a wider panel of markers. While AQP1 and PDZK1 are a good start, they are expressed in other segments of the nephron, so it would be important to check additional markers such as LRP2 or SLC9A3 or others from recent single cell maps of the kidney.

*AR: We have expanded the set of markers and tested 8 additional genes by qPCR. The markers were obtained from recent single cell RNA analyses (Young MD, et al, Science, 2018; Lake BB, et al, Nat Communications, 2019; Sivakamasundari V., et al, bioRxiv, 2017) or a phenotypic characterization of kidney progenitors (Angelotti ML, et al., Stem Cells, 2012) Our phenotypic characterization is now based on 5 markers of kidney progenitor/stem cells (2 assessed by FACS and 3 by qPCR) and 16 markers of differentiated kidney cells. Both populations (KT1 and KT2) express the markers of kidney progenitors and are negative for the majority of markers of differentiated cells, consistent with both cell types being “progenitor cells”. Therefore we removed the definition of “committed epithelial cells” for KT2 throughout the text. The additional experiments helped us to better clarify the identity of the two populations of kidney progenitors.*

*The KT1 population overlaps with the population of scattered tubular progenitors characterized by the Romagnani group (Angelotti et al, Stem Cells, 2012). In fact these cells are CD24/CD133 positive, VCAM1/CD106 low, PDX negative. According to Angelotti´s characterization, these cells mainly localize in the S3 segment of the proximal tubule and can be more rarely found in the distal convoluted tubule and connecting segment, specifically in that portion of the distal convoluted tubule that takes contact with the vascular pole of the glomerulus. Our clones are negative for all markers of PT, DT and glomerulus, except SLC17A3, which is a marker of the S1 segment of the PT. We therefore conclude that KT1 are uncommitted progenitors localized in the convoluted PT. However, we cannot exclude that other, non-tested clones may instead derive from the DT.*

*The KT2 population, instead, expresses proximal tubule markers AQP1, PDZK1 and SLC17A3, but is negative for other markers of the S1 (Cubn, SLC5A12, LPR) and S3 (SLC22A7) segments. For these reasons, we cannot define this population as “committed towards the proximal tubule” and we rephrased our text. However, a recent single cell RNAseq analysis defined in the healthy, adult kidney a population of PT1 cells that showed a gene-expression profile very close to the PT-derived tumors ccRCC and pRCC (Young et al., Science, 2018). Our KT2 cells nicely overlap with this population, which is defined by high expression of VCAM1/CD106 and SLC17A3. In further agreement, KT2 cells show the same mutational profile of ccRCC and pRCC. In addition, we found high expression of the marker of kidney tubule damage KIM1, which is also expressed by kidney tumors. Overall, our data suggest that the KT2 population is a de-differentiated state of the PT1 population defined by Young et al. The de-differentiation might have happened because of tubule damage, in agreement with KIM1 expression and mutational profiles.*

9- While the individuals contributing cells were healthy, they may still harbor pathogenic mutations that would influence mutation process. Since 2-5% of the population may harbor a germline predisposition to cancer, it would be important to perform an annotation of germline variants that could be linked to cancer.

*AR: We thank the reviewer for suggesting a meaningful addition to the paper. We annotated all germline mutations found in 47 genes known to predispose to cancer. We found 841 variants with clinvar annotation, but none was reported as pathogenic or likely pathogenic. We state the lack of germline variants predisposing to cancer in the first paragraph of results and report a table listing the variants with annotation “risk factor”(N=1) “conflicting interpretation of pathogenicity”(N=11) and “uncertain”(N=6) (new Table S1b). All variants are found in heterozygosity.*

**Second round of review**

**Reviewer 1**

I am happy with the revisions and have no further comments

**Reviewer 2**

Their explanations and modifications strongly improved paper clarity and scientific values. Some minor points remain to be addressed in order to further improve the scientific value work:

1- Regarding somatic variant calling, the authors described a method consistent with other literature evidence. Nonetheless, is not completely clear:

non-neutral culture-related mutations. How can these be discarded based on the utilized methodology? It is clear how neutral mutations would result in AF<0.4 even if occurring at first passage, but what about non-neutral ones? Is there any reason to declare they were discarded? Otherwise this should be better specified as methods limitation.

2- Regarding chromosome X and Y mutations. "Chromosome X and Y were excluded from the analysis, because it is impossible to apply the same filtering on AF." I do not understand by AF filters cannot be applied. Even if it is not included in the clustering analysis, I think it will be important to include a table with the information on Chr X somatic variants

3- It would be good to include an annotation of somatic variants based on allele frequencies in large population databases such as Gnomad and dbSNP, as an indication of the rarity of somatic variants

4- Germline cancer predisposing variants. Authors screened germline cancer predisposing mutations based exclusively on ClinVar known pathogenicity, but this is an incomplete because novel pathogenic mutation could be present and never reported in Clinvar. Is would be preferable to annotate the cancer related mutations through a comprehensive assessment, as recommended in the American College of Medical Genetics guidelines [doi: 10.1038/gim.2015.30], in order to avoid possible biases due to ClinVar exclusive utilization. At a very least, any novel LOFs and predicted deleterious missense variants in these genes should be reported.

**Authors Response**

**Point-by-point responses to the reviewers’ comments:**

Reviewer #2: Their explanations and modifications strongly improved paper clarity and scientific values. Some minor points remain to be addressed in order to further improve the scientific value work:

1- Regarding somatic variant calling, the authors described a method consistent with other literature evidence. Nonetheless, is not completely clear:

non-neutral culture-related mutations. How can these be discarded based on the utilized methodology? It is clear how neutral mutations would result in AF<0.4 even if occurring at first passage, but what about non-neutral ones? Is there any reason to declare they were discarded? Otherwise this should be better specified as methods limitation.

*Author reply (AR): The method section did not mention a distinction between neutral and non-neutral variants. However, we agree that it is useful to add a comment about the possibility that non-neutral mutations are included in the lists of somatic variants. This has been added in the methods on “Somatic mutation calling”, on page 25, last paragraph and 26 first lines.*

2- Regarding chromosome X and Y mutations. "Chromosome X and Y were excluded from the analysis, because it is impossible to apply the same filtering on AF." I do not understand by AF filters cannot be applied. Even if it is not included in the clustering analysis, I think it will be important to include a table with the information on Chr X somatic variants

*(AR): The list of somatic variants recovered on X Chromosomes of female donors using our pipeline has been added as Additional file 3.*

3- It would be good to include an annotation of somatic variants based on allele frequencies in large population databases such as Gnomad and dbSNP, as an indication of the rarity of somatic variants

*(AR): The annotation has been added as a new column in Additional file 2. Since our donors are Swedish, the variant frequency has been obtained from a specific database of the Swedish population (SweGen from Ameur et al, 2017*

*https://swefreq.nbis.se/dataset/SweGen/browser)*

4- Germline cancer predisposing variants. Authors screened germline cancer predisposing mutations based exclusively on ClinVar known pathogenicity, but this is an incomplete because novel pathogenic mutation could be present and never reported in Clinvar. Is would be preferable to annotate the cancer related mutations through a comprehensive assessment, as recommended in the American College of Medical Genetics guidelines [doi: 10.1038/gim.2015.30], in order to avoid possible biases due to ClinVar exclusive utilization. At a very least, any novel LOFs and predicted deleterious missense variants in these genes should be reported.

*(AR): Table S1b now also reports variants showing annotation IMPACT=HIGH from SnpEFF http://snpeff.sourceforge.net/VCFannotationformat_v1.0.pdf*

*These include all stop gained, stop lost, frameshift, splice donor/acceptor variants.*
